# Strong philopatry in an estuarine‐dependent fish

**DOI:** 10.1002/ece3.10989

**Published:** 2024-03-18

**Authors:** Koster G. Sarakinis, Patrick Reis‐Santos, Stephen C. Donnellan, Qifeng Ye, Jason Earl, Bronwyn M. Gillanders

**Affiliations:** ^1^ Southern Seas Ecology Laboratories, School of Biological Sciences The University of Adelaide Adelaide South Australia Australia; ^2^ South Australian Museum Adelaide South Australia Australia; ^3^ South Australian Research and Development Institute Aquatic and Livestock Sciences Adelaide South Australia Australia

**Keywords:** *Acanthopagrus butcheri*, Australia, gene flow, philopatry, population structure, single‐nucleotide polymorphisms

## Abstract

Understanding fish movement is critical in determining the spatial scales in which to appropriately manage wild populations. Genetic markers provide a natural tagging approach to assess the degree of gene flow and population connectivity across a species distribution. We investigated the genetic structure of black bream *Acanthopagrus butcheri* across its entire distribution range in Australia, as well as regional scale gene flow across south‐eastern Australia by undertaking a comprehensive analysis of the populations in estuaries across the region. We applied genome‐wide sampling of single‐nucleotide polymorphism (SNP) markers generated from restriction site‐associated DNA sequencing. Genetic structure and potential gene flow was assessed using principal component analyses and admixture analyses (*STRUCTURE*). Using 33,493 SNPs, we detected broad scale genetic structuring, with limited gene flow among regional clusters (i.e. Western Australia, South Australia and western Victoria; and eastern Victoria, Tasmania and New South Wales). This is likely the result of unsuitable habitats, strong ocean currents (e.g. the Leeuwin Current and the East Australian Current), large water bodies (e.g. Bass Strait) and known biogeographical provinces across the continent. Local‐scale genetic structuring was also identified across the south‐eastern Australian estuaries sampled, reflecting that the coexistence of both migratory and resident individuals within populations (i.e. partial migration), and the movement of fish into coastal waters, still results in strong philopatry across the region. Instances of movement among estuaries at this spatial scale were primarily found between adjacent estuaries and were likely attributed to lone migrants utilising inshore coastal currents for movement beyond nearby habitats. Targeting SNP markers in *A. butcheri* at this continental scale highlighted how neither spatial proximity of estuaries nor black bream's ability to move into coastal waters reflects increased gene flow. Overall, our findings highlight the importance of location‐specific management.

## Graphical abstract

Using genome‐wide sequencing of single‐nucleotide polymorphisms, we detected broad scale structuring across the species' entire distribution. We also identified local scale philopatry, supporting the species' estuarine dependency and highlighting the importance of local‐scale management.

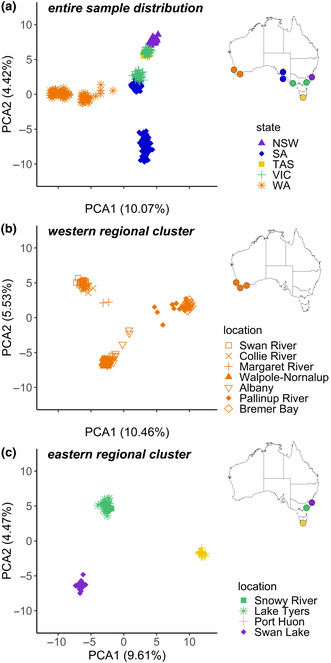

## 1 INTRODUCTION

Animal movement that leads to effective reproduction is a critical migratory process that can structure populations and influence gene flow across a species distribution. As only a few migrants per generation can decrease genetic differentiation between populations (Mills & Allendorf, 1996; Wang, 2004), a change in conditions that leads to an increase in migrants could over time increase gene flow (i.e. connectivity) between two locations to form a single distinct population. Inversely, multiple generation return of populations to a breeding site that leads to reproductive isolation (i.e. philopatry; Secor, 2015) can result in decreased gene flow forming multiple, genetically isolated populations. The potential of such animal movement can have major implications on how we manage overexploited or conservation interest species. Yet, while many marine species have large‐scale distributions, we generally lack an understanding of the structuring and movement patterns of all or large parts of populations, but such information is necessary to reliably evaluate broad scale changes in ecological conditions and support cross‐jurisdictional or transnational management efforts. Overall, historical and contemporary movement among locations, and any resulting gene flow determines the spatial and temporal scales at which management regulations should be implemented.

Genetic markers are natural tags that play a key role in determining population structure and connectivity, providing an alternative, or complement, to artificial tagging techniques such as conventional and telemetry tagging (Cooke & Cowx, 2006). While applied tags can be used to reconstruct individual movements, they are restricted temporally to an individual's life span and the date of tag deployment, whereas genetic markers function on a generational to evolutionary timescale documenting gene flow and genetic differences within and among locations and enable investigating the evolutionary factors that drive this variation (Garvin et al., 2010; Morin et al., 2004; Reis‐Santos et al., 2018).

Nuclear DNA and mitochondrial DNA markers are commonly used for inferring genetic population structure. With the advent of next‐generation sequencing technologies and complexity reduction approaches, genome‐wide sampling of nuclear single‐nucleotide polymorphisms (SNPs) can now be readily applied to model organisms (i.e., extensively studied) and non‐model organisms (Aitken et al., 2004; Garvin et al., 2010). The power of SNP markers lies in their abundance and distribution across genomes (1000s to 100,000s of markers), while also being effective at detecting population structure and connectivity in marine fishes with high levels of dispersal, fish movement and gene flow across broad spatial scales (Anderson & Garza, 2006; Bernatchez et al., 2017; Hall & Beissinger, 2014). Additionally, the power of an increased number of markers can increase the resolution of genetic structure (Sunde et al., 2020).

The black bream, *Acanthopagrus butcheri*, is distributed across southern Australia, ranging from Western Australia in the west to New South Wales in the east, including Tasmania (Norriss et al., 2002). It supports important commercial and recreational fisheries across this range. A long‐lived and slow‐growing species, this sparid matures at approximately 2–4 years of age (28–34 cm total length [TL]) and has a longevity of 32 years (55 cm TL) (Gray, 2008; Izzo et al., 2017; Ye et al., 2013). Due to its tolerance of dramatic shifts in water salinity and temperature, *A. butcheri* can inhabit coastal waters, estuaries and rivers, although it is regarded as an estuarine‐dependent species, requiring estuaries to complete its life cycle (Doubleday et al., 2015; Partridge & Jenkins, 2002). Movement out of estuarine systems can vary among locations, with the coexistence of both migratory and resident life cycles known to occur within the same population (i.e. partial migration; Gillanders et al., 2015; Lack, 1944). However, we are unsure whether fish are moving among estuaries or returning to their estuary of origin. Given the large geographical distribution, spawning periods can vary among estuaries but typically occur during austral spring and summer (Jenkins et al., 2018). Spawning occurs in the upper reaches of streams feeding estuaries (Sakabe et al., 2011; Williams et al., 2012), with eggs hatching ~36–48 h after fertilisation followed by a larval duration of approximately 20–30 days, during which they recruit within estuaries (Roberts et al., 2010). Hybridisation is known to occur with *Acanthopagrus australis* (yellowfin bream) in the species' eastern Australian distribution and is a possible threat to the persistence of southern New South Wales *A. butcheri*. Genetic swamping has been found to occur in coastal lagoons where only ~5% of fish were *A. butcheri*, with the remainder either hybrids or *A. australis* (Farrington et al., 2000; Roberts et al., 2009, 2010; Roberts & Ayre, 2010).

Our understanding of the population structure and gene flow of this estuarine‐dependent species is limited. Population structure studies on *A. butcheri* have focussed mainly on specific sections of either the western or eastern coast of Australia (Chaplin et al., 1998; Gardner et al., 2013; Roberts et al., 2010, 2011). Only one study has covered the species' distribution range but it focused on hybridisation and interspecific gene flow between *A. butcheri* and *A. australis* (Roberts et al., 2009). Therefore, importantly, characterising the population structure of *A. butcheri* across its distribution can provide valuable information on movement among estuaries spread across a continent that is characterised by highly variable climate, rainfall and runoff patterns, as well as fishing effort. Geographical differences in climates and biomes are likely to play a role in the population structure of *A. butcheri* when comparing the connectivity among large, nearby estuaries in subtropical‐temperate south‐eastern Australia to smaller, more geographically isolated, and temporarily open systems found across the Mediterranean climate regions of Western Australia and South Australia. The potential influence of large biogeographical breaks may also be evident (Bennett & Pope, 1953; Waters & Roy, 2003), such as the absence of estuaries across the Great Australian Bight separating Western Australia and south‐eastern Australian estuaries, and the Bass and Investigator Straits separating Tasmanian and Kangaroo Island estuaries, respectively, from those on the mainland.

Population structure, gene flow and inter‐specific hybridisation research on *A. butcheri* has targeted a range of genetic markers, including microsatellites (Gardner et al., 2014; Roberts et al., 2010, 2011; Yap et al., 2000), allozymes (Chaplin et al., 1998; Farrington et al., 2000), and a combination of both nuclear DNA and mitochondrial DNA (Burridge et al., 2004; Burridge & Versace, 2006; Roberts et al., 2009). Inference of population structure has varied based on the sample distribution and genetic marker. Allozymes have reflected a panmictic relationship across Victorian estuaries (~800 km sampling distribution), while mitochondrial DNA and microsatellites highlighted a higher degree of genetic structuring across the same region (Burridge et al., 2004; Burridge & Versace, 2006; Farrington et al., 2000). Allozyme markers also detected genetically distinct populations along the west coast of Australia (Chaplin et al., 1998). Unique marker characteristics may be why our understanding of *A. butcheri* genetic structuring is inconsistent, with allozymes generally having lower resolution compared to mitochondrial DNA and microsatellites (Amiteye, 2021; Liu & Cordes, 2004). A more comprehensive and contemporary approach across the entire distribution range and using genome‐wide markers is expected to shed light on the population structure and degree of gene flow, together with insights into the impacts of environmental and biogeographical boundaries at both regional and continental scales.

Here we use genome‐wide sampling of SNP markers generated from a complexity reduction method—restriction site‐associated DNA sequencing, to infer the population structure of *A. butcheri* on both a broad and local scale. Specifically, we harnessed the power of this cost‐effective next‐generation sequencing to investigate (1) how *A. butcheri* is genetically structured across its distribution range, and (2) the degree of gene flow at the local scale in south‐eastern Australia where sampling of individual estuaries was geographically comprehensive. We hypothesise that *A. butcheri* would show limited gene flow at a regional scale, reflecting the species' estuarine dependency. Furthermore, we hypothesise that the inconsistent freshwater outflow across southern Australia, along with known biogeographical boundaries will further limit movement of *A. butcheri* at both regional and continental scales.

## 2 MATERIALS AND METHODS

### 2.1 Field sampling and sample preparation

**FIGURE 1:**
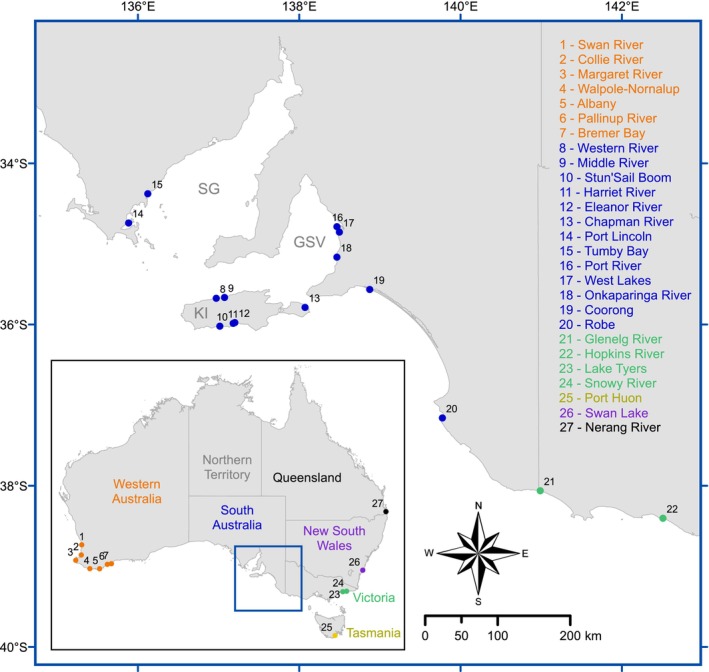
Map of South Australia, with an inset of Australia showing all 27 *Acanthopagrus butcheri* and *Acanthopagrus australis* capture locations. Samples were collected from Western Australia (orange), South Australia (blue), Queensland (black), New South Wales (purple), Victoria (green) and Tasmania (yellow). Map of South Australia also highlights Kangaroo Island (KI), Spencer Gulf (SG) and Gulf Saint Vincent (GSV).

Samples of *Acanthopagrus butcheri* were collected from estuaries across the species' entire distribution range (i.e. from Western Australia to New South Wales) between March 2020 and January 2022 (Figure 1, Table 1). Samples were collected from key estuaries in South Australia and across southern Australia through collaborative efforts with interstate government agencies and angling associations, with not only rod and line the primary method of capture but also seine and gill netting in permitted locations (The University of Adelaide animal ethics, approval S‐2020‐069). Whole fish were collected, aiming for 20 fish per estuary, although a non‐fatal tail clipping technique was used where community‐enforced catch‐and‐release regulations applied (e.g. Port Lincoln and Onkaparinga River, South Australia). To evaluate potential genetic variation over time, a collection of historical frozen liver samples from Western Australia dating back to 1996 were included in our analyses (Chaplin et al., 1998). Where possible, each fish was measured for total length to the nearest millimetre and weighed to the nearest gram. A sample of soft muscle tissue (~1 cm^3^) and a tail clipping (2–5 cm long) were collected from each fish and individually preserved in 100% ethanol.

**TABLE 1:** *Acanthopagrus butcheri* capture locations with corresponding Australian state, regional cluster and sub‐cluster classifications identified using Principal Component Analyses (PCA), along with collection year and sample sizes.

State	Capture location		Collection year	Sample Size	Regional cluster	Sub‐cluster
Western Australia (WA)	1	Swan River	2020	19	Western	–
	2	Collie River	1996	16		
	3	Margaret River	1996	3		
	4	Walpole‐Nornalup	2020	19		
	5	Albany	2020	19		
	6	Pallinup River	1996	15		
	7	Bremer Bay	2020	19		
South Australia (SA)	8	Western River	2020	18	Southern	northKI
	9	Middle River	2020	16		
	10	Stun Sail Boom	2020	18		southKI
	11	Harriet River	2020	21		
	12	Eleanor River	2020	18		
	13	Chapman River	2020	9		
	14	Port Lincoln	2021	15		northSA
	15	Tumby Bay	2021	11		
	16	Port River	2021	8		
	17	West Lakes	2021	25		
	18	Onkaparinga River	2021	7		
	19	Coorong	2020–2021	21		southSA
	20	Robe	2020–2021	20		
Victoria (VIC)	21	Glenelg River	2021	22		westVIC
	22	Hopkins River	2021	21		
	23	Lake Tyers	2021–2022	31	Eastern	–
	24	Snowy River	2021	4		
Tasmania (TAS)	25	Port Huon	2021	21		
New South Wales (NSW)	26	Swan Lake	2020	23		

### 2.2 SNP genotyping and data processing

Tissue samples were submitted for DNA extraction and DArTseq™ 1.0 genotyping at Diversity Arrays Technology PL, Canberra, Australian Capital Territory, Australia (medium‐density, 1.2 million reads per sample). DArTseq™ represents a combination of DArT genome complexity reduction methods and next generation sequencing platforms, targeting the predominately active areas in the genome (low copy fragments) containing the most useful information (Kilian et al., 2012). DNA samples were processed in restriction enzyme digestion/ligation reactions using a combination of the *Pst*I/*Sph*I restriction enzymes. Ligated fragments were PCR amplified as described by Kilian et al. (2012) and Mahony et al. (2020) for single end sequencing for 77 cycles on an Illumina Hiseq2500. A matrix of SNP genotypes for each individual was received from DArT as a 1‐row binary score for each locus.

The raw SNP data underwent a filtering pipeline similar to Junge et al. (2019). Four filters were applied, namely (1) removal of loci with <95% reproducibility, (2) retaining loci with <20% missing data, (3) removing secondary loci (e.g. retaining one of two or more separately recorded SNP loci for the same fragment), and (4) removing monomorphic loci (*DArTr* package in *R*; Gruber et al., 2018). We retained loci with <20% missing data, given the large number of loci, with no individuals removed due to missing data.

To identify potential hybrids that could influence our interpretations of genetic structuring, the closely related species *A. australis* was sampled from the Nerang River in southern Queensland (*n* = 10, Figure 1, Table S1), north of the zone of hybridisation with *A. butcheri* (Roberts et al., 2010). Potential hybrids between *A. butcheri* and *A. australis* were identified using a Principal Component Analysis (PCA). This was undertaken for samples from all capture locations to enable the detection of potential expansion of the hybridisation zone via range shifts of *Acanthopagrus* spp. (Pecl et al., 2014). PCA scores were generated from the filtered SNP data and plotted (*adegenet* and *ggplot2* packages in *R*; Jombart, 2008; Wickham, 2009). Hybrids were identified as falling between the two species clusters, either evenly positioned between (*F*_1_ hybrid) or partially (*F*_2_ or backcrossed hybrid).

With many fisheries‐based studies relying on trawling and manual netting procedures to collect samples (Reis‐Santos et al., 2018; Sarakinis et al., 2022), assessing relatedness within sample sets is essential to accurately infer population structure and connectivity. There is the possibility that fish caught within an estuary on the same day or from the same net haul are closely related. As a mixture of collection types were used in this study (e.g. net and/or rod, Table S1), and relatedness can influence the inference of population structure, the assumption of random sampling was validated using an identity‐by‐descent analysis (*SNPRelate* package in *R*; Zheng et al., 2012). Kinship coefficient values were generated using the KING (robust) method of moment for each possible pairing of *A. butcheri*, both within and among capture locations; with a kinship coefficient of ≥0.25 representing a sibling–sibling or parent–offspring relationship (Sun & Dimitromanolakis, 2014). Closely related individuals were removed, retaining only a single individual from highly related groups. All analyses were performed in *R* (R Core team, 2023).

### 2.3 Population structure

Population structure was investigated with two clustering approaches, PCA and admixture analysis to determine whether *A. butcheri'*s distribution comprises isolated breeding populations. Ten PCA scores were generated to determine which combination of PCs identified genetic clusters. Pairwise fixation index (FST) values were also calculated as a genetic distance matrix to assess genetic variance between locations (*StAMPP* package in *R*; Pembleton et al., 2013). Clusters identified by both approaches that were indicative of potential genetic population structuring were independently refiltered through the pipeline for subsequent analyses to retain as many SNPs as possible (i.e. SNP filters 1 to 4 applied to selected individuals using a subset of the raw data) (Table S2).

For admixture modelling, *STRUCTURE* 2.3.4 (Pritchard et al., 2000) was used to determine the true number of genetic populations (*k*). Model parameters included a burn‐in of 50,000 iterations, 100,000 Markov Chain Monte Carlo (MCMC) simulations, and 10 replicates for each predicted *k*. The range of predicted *k* tested was based on the number of capture locations present in the particular dataset analysed (Table 1). Initial output variance determined whether model parameters were adjusted. The true number of genetic populations detected for each model was based on the log likelihood of all predicted *k* (lnP(*k*)), the highest delta *k* (Δ*k*) value determined using the Evanno method (Evanno et al., 2005), and investigating structure plots for each predicted *k* using *StructureSelector* (Li & Liu, 2017). When structure analysis results (i.e. Δ*k* values, lnP(*k*) values, and structure plots) revealed inconsistencies in predicted *k*, additional admixture models were run in a hierarchical approach to validate finer‐scale structuring and *k*.

As sampling sites outside of South Australia were not as exhaustive (lower number of capture locations and absence of sampling of adjacent estuaries), population sub‐structuring detected may also be consistent with isolation by distance. Therefore, we tested for isolation by distance within each of the Western Australian capture locations and the southern New South Wales and eastern Victoria locations. Pairwise distance values (km) were measured manually as the closest distance by water between each capture location, using *Google My Maps*. Using the FST pairwise matrix generated for each *A. butcheri* capture location, the relationship between the geographical distance matrix and genetic distance matrix was assessed using a Mantel test in *GenAlEx 6.5* (Peakall & Smouse, 2012), where a positive correlation indicated geographical isolation by distance. *Acanthopagrus butcheri* from Tasmania were excluded from this test due to the large oceanic barrier formed by Bass Strait separating them from the mainland locations (York et al., 2008).

## 3 RESULTS

A total of 469 samples were submitted for genotyping, including 459 *A. butcheri* and 10 *A. australis*. Sample extract quality control at DArT allowed for 439 *A. butcheri* and 10 *A. australis* to be retained for SNP genotyping (Table S1). The 20 low‐quality samples not genotyped were historical liver material that had been in prolonged storage. The genotype matrix contained 33,493 SNP loci with all individuals and 15,012 loci retained after data filtering (Table S2). We were unable to secure contemporary samples from the same locations as the historical samples (Table S1), but our findings showed genetic similarities between historical and contemporary *A. butcheri* from adjacent sites in WA. Considering the lack of differences among samples collected in 1996 and 2020–2021, all samples were subsequently analysed together for the FST analysis.

### 3.1 Species hybridisation and relatedness

In the PCA performed to identify potential hybrids, no hybrids were detected with no individuals falling between the two distinct species clusters (Figure S1). *Acanthopagrus australis* samples were not included in any subsequent analyses and the raw SNP data were refiltered through the pipeline.

In terms of relatedness between individuals, the average kinship coefficient was −0.279 ± 0.228. Four pairs of samples showed kinship coefficient values greater than 0.25 (Table S3, Figure S2). Each of the four pairs comprised individuals captured from the same location, namely two individuals each from Port Huon (Tasmania), Harriet River (South Australia), Chapman River (South Australia) and Swan Lake (New South Wales). An individual from each pair was removed from all subsequent analyses.

### 3.2 Broad scale population structure

**FIGURE 2:**
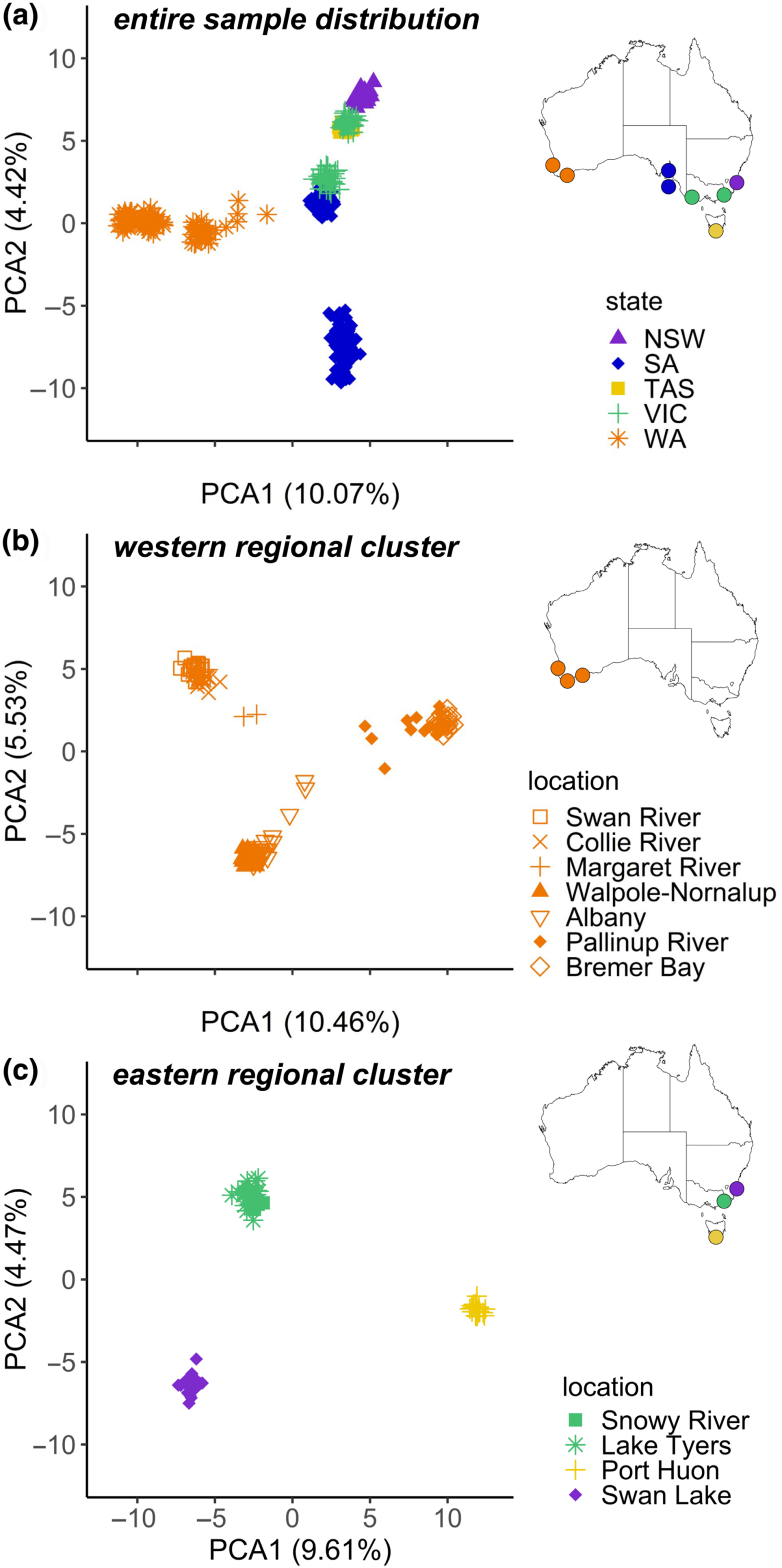
Principal component analysis (PCA) of the (a) entire *Acanthopagrus butcheri* sample distribution, (b) western cluster, and (c) eastern cluster. Data are grouped by Australian states, including Western Australia (WA, orange), South Australia (SA, blue), New South Wales (NSW, purple), Victoria (VIC, green), and Tasmania (TAS, yellow). Data are represented as bivariate plots using PCA1 and PCA2 scores as the axes.

The PCA for *A. butcheri* from across the sampling range identified three regional genetic clusters (Figure 2a, Table 1). The western cluster, which included all fish from Western Australia was separated by PC1 from the remaining samples from eastern Australia. Among the remaining samples, three clusters were separated by PC2, with (1) an eastern cluster comprising samples from eastern Victoria, New South Wales and Tasmania, (2) a southern cluster comprising samples from mainland South Australia and western Victoria, and (3) another southern cluster of *A. butcheri* from Kangaroo Island. However, given their spatial proximity, both southern clusters were treated as one regional cluster for subsequent analyses. The data for each regional cluster were refiltered through the pipeline independently and subject to subsequent PCAs. Both the western cluster (Western Australia) and the eastern cluster (eastern Victoria, New South Wales, Tasmania) showed further separation driven by capture locations (Figure 2b,c). Isolation by distance tests (between individuals) were run for each of the western and eastern clusters (excluding Tasmania), both of which revealed positive correlations in both Mantel tests (*R* ^2^ = .48 and *R* ^2^ = .51 respectively).

**FIGURE 3:**
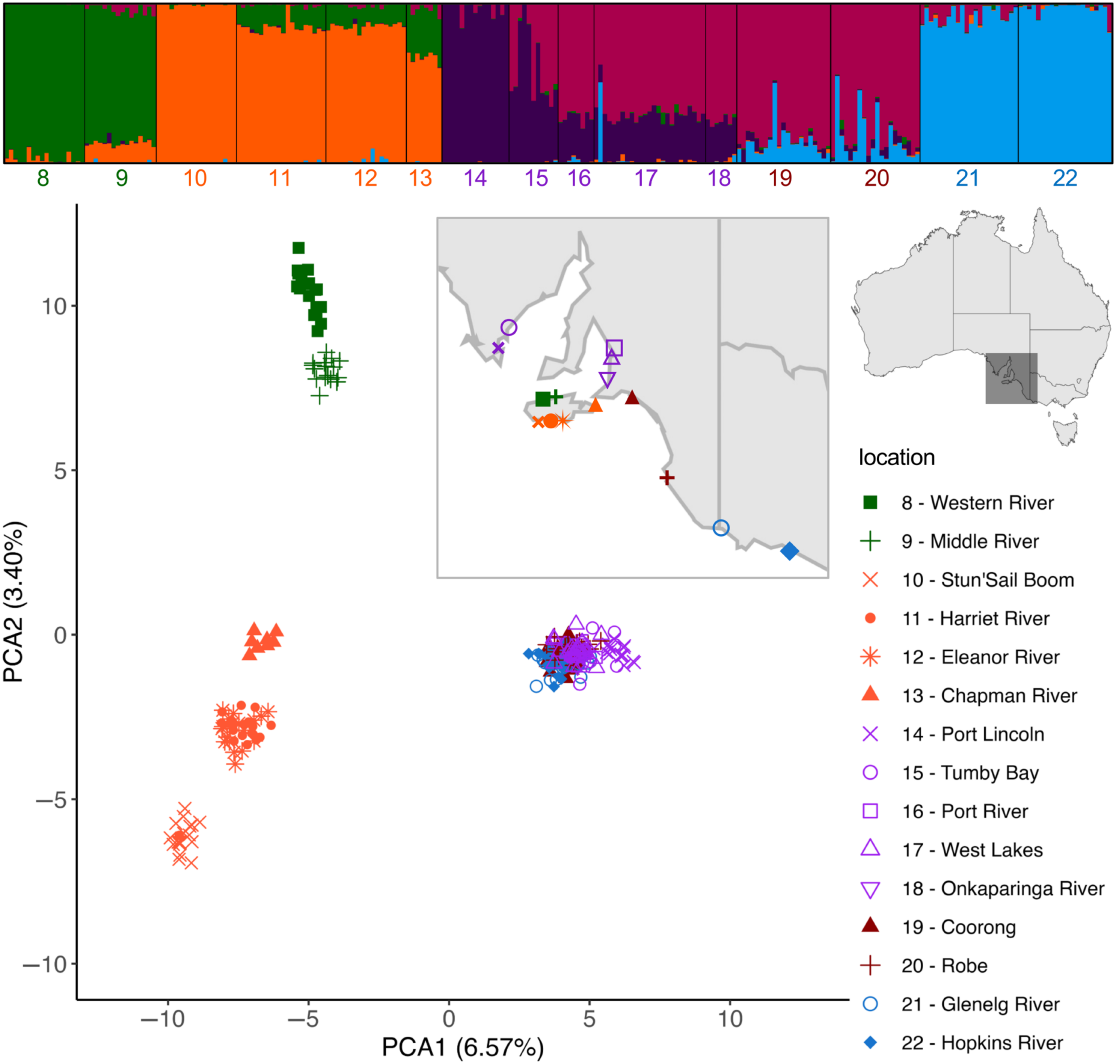
Structure plot (above) and Principal Component Analysis [PCA] plots (below) for the southern regional cluster of *Acanthopagrus butcheri*. Capture locations identifiable by colour, shape and number both in the legend and on the map. Colour assignments are based on the dominant cluster each location is predicted to be a part of in their corresponding structure and PCA plot (i.e. unique to each sub‐cluster).

**FIGURE 4:**
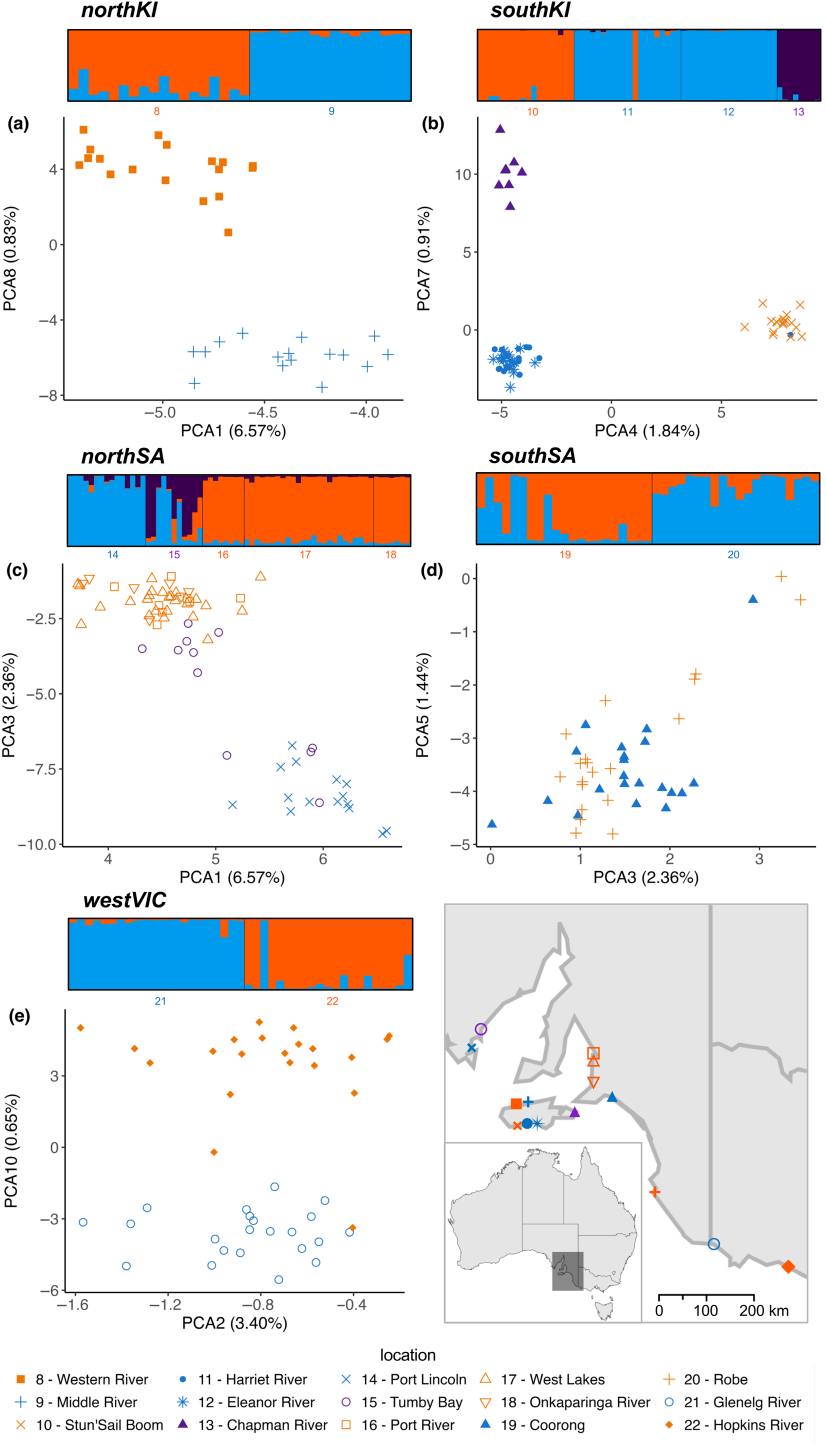
Structure plots (above) and Principal Component Analysis [PCA] plots (below) for the five sub‐clusters of the southern regional cluster of *Acanthopagrus butcheri* collected in South Australia and eastern Victoria, including sub‐clusters (a) northKI, (b) southKI, (c) northSA, (d) southSA and (e) westVIC. Each capture location is identifiable by colour, shape, and number both in the legend and on the map. Colour assignments are based on the dominant cluster each location is predicted to be a part of in their corresponding structure and PCA plot (i.e. unique to each sub‐cluster). PCA plots were created using two out of the ten PC scores generated that best separated the sub‐clusters.

As the southern cluster comprised the most geographically close estuaries sampled, we investigated population structure within this region in further detail. Pairwise fixation indices for the southern cluster showed a clear pattern of higher genetic differentiation between mainland South Australia and Kangaroo Island (all FST >0.10), as well as among the Kangaroo Island capture locations (Table S4). Fish from Stun'Sail Boom (Kangaroo Island) exhibited consistent higher genetic differentiation (FST >0.15, and up to 0.22 between Stun'Sail Boom and Port Lincoln), except for the two closest estuaries (i.e. Harriet River and Eleanor River, FST = 0.09 and 0.10 respectively). FST values among the mainland capture locations from Port River (South Australia) to Hopkins River (Victoria) were all ≤0.05 and down to 0.0017 (Port River and West Lakes, South Australia). The southern regional cluster PCA revealed discrete genetic clusters comprising *A. butcheri* from mainland Australia, northern Kangaroo Island and southern Kangaroo Island capture locations (Figure 3). Eight of ten PCs showed evidence of subtle, finer scale structure, which further separated these capture locations into five sub‐clusters (Figure 4). There was separation between the north and south coast of Kangaroo Island (sub‐clusters northKI and southKI), as well as between the two major gulfs of South Australia and the eastern South Australian coastline (sub‐clusters northSA and southSA respectively). The fifth sub‐cluster comprised *A. butcheri* from the western Victorian capture locations (sub‐cluster westVIC) (Table 1). Subsequent analyses for admixture at local scales were conducted on the southern regional cluster.

### 3.3 Local scale population structure

An admixture model was run on the entire southern cluster, with the exploratory parameters producing acceptable output statistics. By applying the Evanno method (i.e. highest Δ*k*) (Evanno et al., 2005), five distinct populations (*k* = 5) were identified within the southern regional cluster that matched those identified using PC scores though there was also a secondary Δ*k* peak at *k* = 8 (Figure 3, Figure S3a). Furthermore, lnP(*k*) values plateau at *k* = 8 (Figure S3b), with the structure plots of both predicted *k* showing similar grouping of capture locations, with additional clusters between Spencer Gulf and Gulf St Vincent (i.e. sub‐cluster northSA) and a single individual from West Lakes appearing to have ancestry unique from all capture locations (Figure S4). This inconsistency suggests there may be hierarchical structure and, therefore, further admixture models were run for all five sub‐clusters previously identified, including the additional clusters predicted here that are within sub‐cluster northSA.

Admixture modelling for the sub‐clusters reinforced the patterns observed in the PCAs, with *k* = 2 detected for sub‐cluster southKI (Figure 4a) based on both Δ*k* (Figure S5a) and lnP(*k*) values (Figure S5b) with a clear separation between capture locations in the corresponding structure plots (Figure S6). Three populations were identified for sub‐cluster southKI (*k* = 3, Figure 4b, Figure S7), with structure plots consistently identifying one individual from Harriet River as a likely migrant from Stun'Sail Boom (approximately 24 km apart) (Figure S8). Three populations (*k* = 3) were detected for sub‐cluster northSA based on lnP(*k*) values (Figure 4c, Figure S9b), showing a separation within Spencer Gulf (between Port Lincoln and Tumby Bay, with some Tumby Bay samples showing Port Lincoln ancestry and Gulf St Vincent ancestry), and between Spencer Gulf and Gulf St Vincent (Port River, West Lakes, Onkaparinga River; see Figure 1). However, Δ*k* detected *k = 4* (Figure S9a), with the corresponding structure plots identifying the same individual from West Lakes previously identified that appeared to have ancestry unique from all capture locations (Figure S10). Two populations were detected in sub‐cluster southSA based on Δ*k* values (Figure S11a), with structure plots revealing some Coorong samples with Robe ancestry (Figure 4d, Figure S12). However, lnP(*k*) indicated *k* = 1 (Figure S11b) and considering the Evanno method cannot predict *k* = 1, as well as the similarities of both capture locations using PC scores, it is likely that this is a single population (Figure 4d). Two populations were identified for sub‐cluster westVIC (Figure 4e) based on both Δ*k* values (Figure S13a) and lnP(*k*) values (Figure S13b), with structure plots revealing a lone migrant sampled from the Glenelg River that had an ancestry consistent with it originating from the Hopkins River (Figure 4e, Figure S14).

## 4 DISCUSSION

Using a continental‐scale comprehensive sampling of *A. butcheri* across its 3200‐km longitudinal range, we highlight the power of genome wide SNP data to understand population structure and connectivity patterns for estuarine‐dependent species. Genetic patterns identified in this study across *A. butcheri's* distribution range are likely the result of the interplay between the species' life history and large‐scale marine biogeographical barriers and local‐scale habitat distribution.

### 4.1 Broad scale structure

The genetic differentiation among regional clusters is likely attributable to geographical barriers. The absence of suitable habitats for *A. butcheri* between Western Australia and South Australia (i.e. the absence of freshwater outflows and estuaries) would explain the genetic isolation between the western and southern clusters (~2150 km apart) (Figures 1 and 2a). Studies that targeted allozyme and microsatellite markers have shown similar isolation across both Western Australia (Chaplin et al., 1998) and South Australia (Burridge et al., 2004). In particular, barriers such as the Great Australian Bight limit movement of estuarine‐dependent species (e.g. Hammer et al., 2021) and support our findings on the low likelihood of movement or gene flow at this distance (Hammer et al., 2021). Gene flow at such a broad geographical scale is expected to be relatively low compared to marine fish that utilise ocean currents for egg and larval dispersal (Bennett et al., 2017; Islam et al., 2022). It is unlikely that *A. butcheri* could utilise such a dispersal strategy given that they spawn in the upper reaches of estuaries, with spawning events dependent on freshwater flow and occurring away from any coastal currents (Roberts et al., 2010; Williams et al., 2012). Gene flow across marine habitats associated to individual movements is not however ruled out for mature *A. butcheri* and may be contributing to the albeit limited gene flow seen at larger distances (i.e. 100 s kms), as seen in other species (Cheng et al., 2015; Tan et al., 2022). Overall, the need for estuarine conditions for spawning and subsequent egg and larval survival (Jenkins et al., 2018; Williams et al., 2020) is reflected in the overall philopatry we have identified, with connectivity among estuaries most likely a result of adult fish movements. The absence of suitable habitats limiting gene flow has also been found in reef fish, where open water between rocky or coral reefs restricts gene flow and drives population structuring (Ducret et al., 2022; Torres‐Hernandez et al., 2022). Furthermore, as *A. butcheri* have been shown to move out of estuarine systems through tagging, telemetry and otolith chemistry (Gillanders et al., 2015; Hindell et al., 2008; Hoeksema et al., 2006), the high genetic structuring found best reflects philopatry (i.e. individuals returning to capture locations) rather than year‐round residency.

The genetic distinction between *A. butcheri* sampled from western Victoria and eastern Victoria aligns with the recognised boundaries of marine biogeographical provinces in southern Australia, namely the Flindersian and Peronian provinces respectively (Bennett & Pope, 1953; Li et al., 2013; Waters & Roy, 2003). This boundary coincides with species distribution limits, as well as population structuring for several species (Colgan, 2016), including an east–west divergence previously identified for *A. butcheri* and other sparids such as snapper, *Chrysophrys auratus* (Bertram et al., 2023; Burridge & Versace, 2006). The genetic differentiation present across Victorian locations is explained by the presence and convergence of strong ocean currents found across south‐eastern Australia (i.e. Leeuwin Current and East Australian Current [EAC]) creating a break in gene flow. Similar barriers reducing gene flow include the Kuroshio current along southern China (Gu et al., 2022; Islam et al., 2022), the currents along eastern and southern Africa (Gaylord & Gaines, 2000; Reid et al., 2016), as well as the lower Congo River rapids (Kurata et al., 2022; Markert et al., 2010). Ocean currents would also explain the genetic similarities found within biogeographical provinces, such as the eastern cluster (Peronian province, Figure 2a), where the EAC has previously been known to assist gene flow and movement of marine species, including oysters (*Saccostrea glomerata*, O'Hare et al., 2021), reef fish (*Parma microlepis*, Curley & Gillings, 2009) and snapper (*C. auratus*, Sumpton et al., 2008). However, when the eastern cluster is isolated from the remaining capture locations, there is clear genetic differentiation across *A. butcheri* from each Australian state (Figure 2c). This differentiation was also shown within the regional clusters, with capture locations mirroring their geographical orientation within both the western cluster (Figure 2b) and southern cluster (Figure 3). Genetic structure mirroring geography has also been shown in populations of water fleas (*Daphnia magna*, Fields et al., 2015), scallops (Pecten maximus, Vendrami et al., 2019) and terrestrial mammals (de Jong et al., 2020; Kominakis et al., 2021).

Individual‐scale tests show isolation by distance within both the western cluster and eastern cluster, suggesting connectivity among nearby locations and restricted gene flow with increasing distances at this spatial scale. However, given the geographically discontinuous sampling across both regional clusters, fish movement may still occur between capture locations in adjacent estuaries. Therefore, increased sampling effort (i.e. sampling adjacent estuaries) is recommended to further resolve population structure and investigate the degree of gene flow at the local scale along the eastern and western Australian coastlines.

Admixture modelling of the southern cluster revealed two genetically distinct populations: *A. butcheri* captured from mainland Australia and those from Kangaroo Island. The clear separation of locations is likely attributed to the water bodies between them (i.e. Investigator Strait and Backstairs Passage) limiting fish movement and gene flow. The absence of coastline and/or suitable depth range between habitats has been shown previously as a form of genetic isolation (Nordahl et al., 2019; Spies, 2012; Volk et al., 2021), and coupled with the strong ocean currents and habitat suitability, is likely to be driving genetic differentiation at this spatial scale.

### 4.2 Local scale structure

Independent admixture models of southern sub‐clusters revealed genetic differentiation and subtle, fine‐scale structuring. Gene flow was present between spatially close collection locations (i.e. adjacent estuaries), including Harriet River and Eleanor River identified as a single genetic population (Figure 4b), with estuary mouths only ~2 km apart. ‘Estuary‐hopping’ has been observed previously for *A. butcheri* (Burridge & Versace, 2006; Chaplin et al., 1998; Gillanders et al., 2015) and shows their ability to inhabit coastal waters while still reflecting some form of estuarine dependence. Nonetheless, cases of genetic differentiation were found between adjacent locations, including Middle River and Western River on Kangaroo Island (9 km apart) (Figure 4a). Relative to capture locations on mainland Australia, Kangaroo Island has many small estuaries that undergo seasonally driven flushing events with intermittent openings to the sea that likely limit local scale fish movement (Rumbelow et al., 2010). Genetic differentiation at a local scale shows that the proximity of locations is not a consistent factor driving connectivity in this species. Reduced gene flow influenced by differences among habitats (e.g. freshwater inflow, water temperature and salinity) has been found previously in other species that use estuaries throughout their life cycles (Hollenbeck et al., 2019; O'Leary et al., 2021). Although estuary‐hopping may be common in some locations, appropriate assessments of estuary geography and characteristics (e.g. systems closed for extended periods) are required before generally assuming gene flow between adjacent locations (Lassauce et al., 2022; Le Moan et al., 2016). Overall, the predicted increase in estuary closures in response to climate change across Western Australia and South Australia relative to eastern Australia (Hallett et al., 2017) is likely to further contribute to the limited gene flow present within the western and southern clusters. Furthermore, in light of the expected increase in estuarine closures associated with global change in southern Australia, evaluations of gene flow and population structure of estuarine populations over time are recommended.

In some cases, adjacent estuaries that were genetically distinct, showed low‐level connectivity in the form of lone migrants, where an individual caught from one location was shown to have the genetic ancestry of *A. butcheri* captured elsewhere. This small number of migrants further demonstrates the variation in this species' movement and ability to partially migrate (Gillanders et al., 2015). Migrant exchange via unidirectional flow has been shown in freshwater species, particularly from rivers into lake populations (Bernas et al., 2021; Erin et al., 2019; Roman et al., 2018), as well as between discrete marine populations (Lassauce et al., 2022; Velasco‐Montoya et al., 2022). Investigating genetic variation on a temporal scale across the southern cluster could determine the influence of these lone migrants on the stability of population structuring and connectivity.

Gene flow at greater distances across the southern cluster was revealed between collection locations >200 km apart (e.g. Coorong and Robe), with structure analyses and PC scores predicting a single genetic population (Figure 4d). The presence of seasonally driven inshore currents along the coastline between Robe and the Coorong may have assisted *A. butcheri* movement between these locations (Middleton & Bye, 2007). Seasonal currents may have contributed also to the movements of a lone migrant from Glenelg River to Hopkins River (~191 km apart), considering the south‐easterly flow of the Leeuwin Current during winter, and partial reversal over summer (Li et al., 2013; Waters & Roy, 2003), as used by other sparids (Gardner et al., 2022). Both estuary proximity and inshore currents likely have influenced gene flow in *A. butcheri* from Tumby Bay. This capture location, although identified as a distinct genetic population included migrants originating from Port Lincoln (~57 km away) and similarities with the Gulf St Vincent population (~923 km away from Port River) (Figure 4c). Movement between the two Spencer Gulf locations would not be surprising, given their spatial proximity, although potential movement between the South Australian gulfs would likely be attributed to seasonally driven inshore currents (Middleton & Platov, 2003). Estuaries are present along the coastline of both gulfs, although few are spatially close (e.g. no viable habitats or freshwater outflows along the western coastline of Yorke peninsula) (Rumbelow et al., 2010). Therefore, even with *A. butcheri* estuary‐hopping to adjacent locations, long distance movement would be required for fish to migrate between both South Australian gulfs. Additional sampling along the coastline between Tumby Bay and Gulf St Vincent may be needed to provide greater insight into their connectivity and gene flow associated with regional oceanographic conditions and predicted residual currents between both South Australian gulfs (Rogers et al., 2021).

The presence of interspecific hybrids can influence inferences of population structure. While we tested our data for the presence of hybrids both outside and within the known hybridisation zone at Swan Lake, New South Wales (Farrington et al., 2000; Roberts et al., 2009; Roberts & Ayre, 2010), their absence was not consistent with previous findings of potential genetic swamping within *A. butcheri* populations, as well as predicted range shifts of *Acanthopagrus* spp. (Pecl et al., 2014; Roberts et al., 2010). Further investigations into the extent of hybridisation with increased sampling efforts across New South Wales could explore if the geography of *Acanthopagrus* interspecific hybridisation is changing and whether the impacts of genetic swamping may have stabilised over time (Mandeville et al., 2017).

Our study highlights strong philopatry in this estuarine‐dependent species, with cases of gene flow more present between adjacent estuaries and the limited movement identified at greater distances likely influenced by lone migrants and/or assistance from inshore currents. The geographical isolation found at a broader scale suggests the presence of geographical barriers, most likely in the form of a lack of suitable habitat, ocean currents and large water bodies that drive the formation of biogeographical provinces across southern Australia. Strong philopatry at both a broad and local scale stresses the importance of location‐specific management. Targeting SNPs in *A. butcheri* provides an example of how neither spatial proximity of estuaries nor a fish's movement into coastal waters (i.e. partial migration) is a reflection of gene flow among estuaries.

## AUTHOR CONTRIBUTIONS

**Koster G. Sarakinis:** Conceptualization (equal); data curation (lead); formal analysis (equal); investigation (lead); project administration (lead); visualization (lead); writing – original draft (lead); writing – review and editing (equal). **Bronwyn M. Gillanders:** Conceptualization (equal); funding acquisition (lead); supervision (lead); writing – review and editing (equal). **Jason Earl:** Conceptualization (equal); funding acquisition (equal); supervision (equal); writing – review and editing (equal). **Patrick Reis‐Santos:** Conceptualization (equal); data curation (equal); formal analysis (equal); investigation (equal); supervision (equal); visualization (equal); writing – review and editing (equal). **Stephen C. Donnellan:** Conceptualization (equal); formal analysis (equal); investigation (equal); validation (equal); writing – review and editing (equal). **Qifeng Ye:** Conceptualization (equal); funding acquisition (equal); supervision (equal); writing – review and editing (equal).

## CONFLICT OF INTEREST STATEMENT

The authors declare no conflict of interest.

## Supporting information


Appendix S1.

## Data Availability

The data that support the findings of this study are openly available in figshare at https://figshare.com/articles/dataset/Raw_data_for_Strong_philopatry_in_an_estuarine‐dependent_fish_/24752010.
