# Supplementary material for: Strong philopatry in an estuarine‐dependent fish
Source: Ecol Evol. 2024 Mar 18;14(3):e10989. doi: 10.1002/ece3.10989 (PMC10945236; doi:10.1002/ece3.10989)
Supplement: Supplementary file 1 — Appendix S1. [file ECE3-14-e10989-s001.pdf]

Appendix:
Strong philopatry in an estuarine-dependent fish

Koster G. Sarakinis[1*], Patrick Reis-Santos[1], Stephen C. Donnellan[2], Qifeng Ye[3], Jason Earl[3], Bronwyn M. Gillanders[1]

[1]Southern Seas Ecology Laboratories, School of Biological Sciences, The University of Adelaide, SA 5005, Australia
[2]South Australian Museum, North Terrace, Adelaide, SA 5000, Australia
[3]South Australian Research Development Institute Aquatic Sciences, Adelaide, West Beach, South Australia 5024, Australia

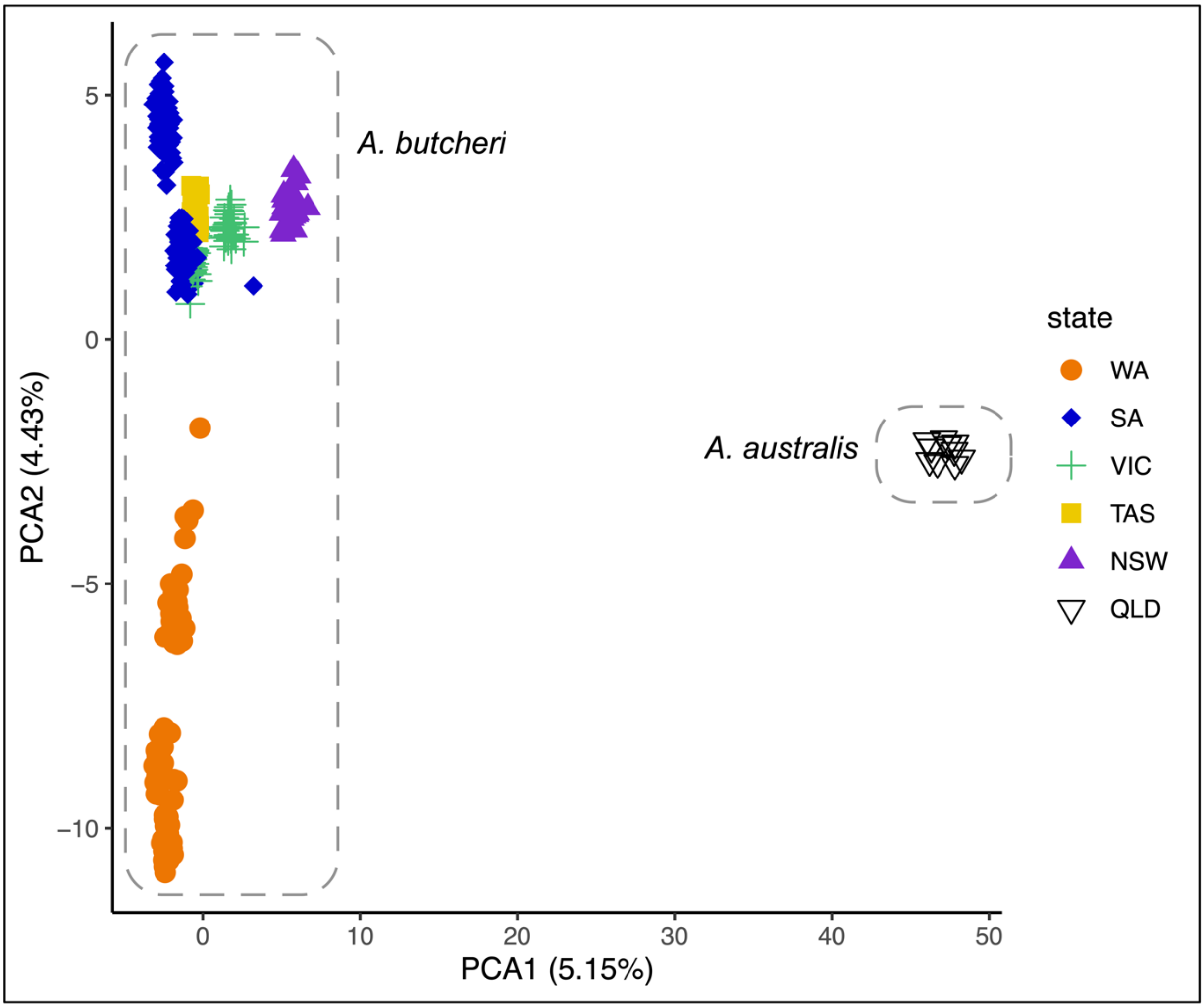

Figure S1. Principal Component Analysis (PCA) of the entire sampling distribution, including both *A. butcheri* and *A. australis*. Data are grouped by Australian states, including Western Australia (WA, orange), South Australia (SA, blue), Queensland (QLD, black inverted triangle , *A. australis*), New South Wales (NSW, purple), Victoria (VIC, green), and Tasmania (TAS, yellow). Data are represented as a bivariate plot using PCA1 and PCA2 scores.

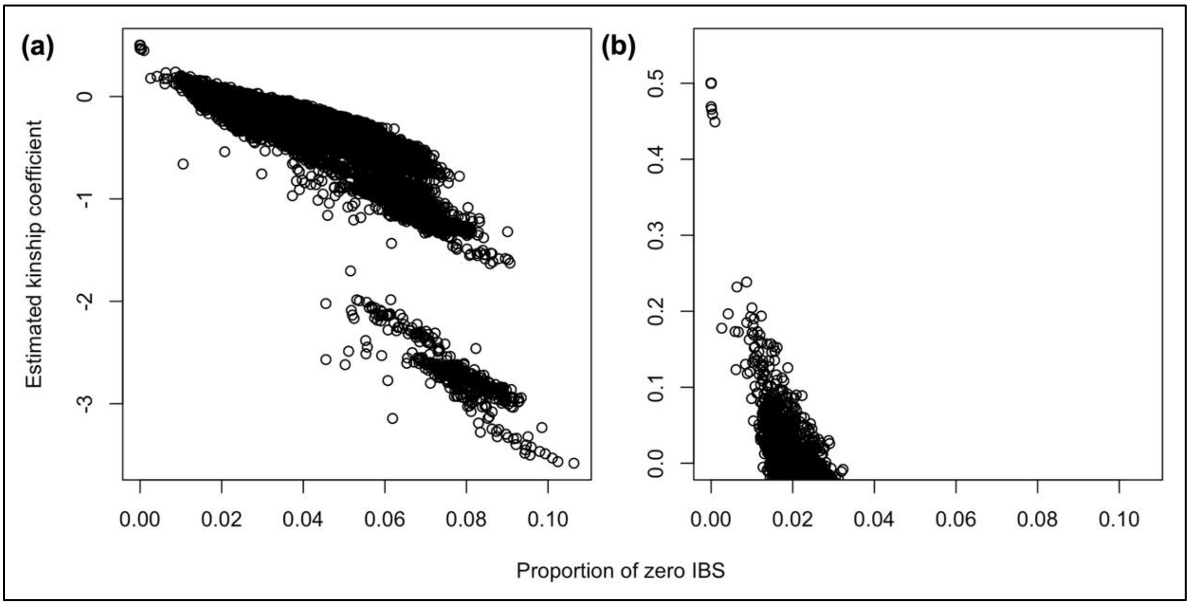

Figure S2. Identity by descent analysis of *A. butcheri* and *A. australis* samples using the KING method of moment. Each data point represents a comparison test between two individuals, with the proportion of SNPs with zero identity-by-state (IBS) plotted against the estimated kinship coefficient. Plots include (a) the entire sample distribution and (b) sample pairings with estimated kinship coefficient values greater than 0.

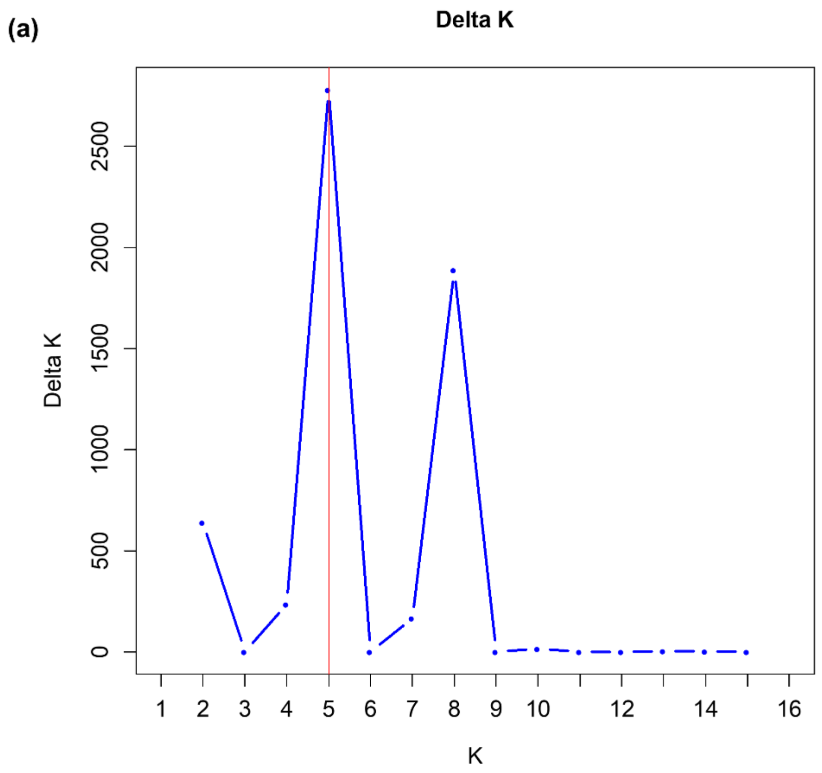

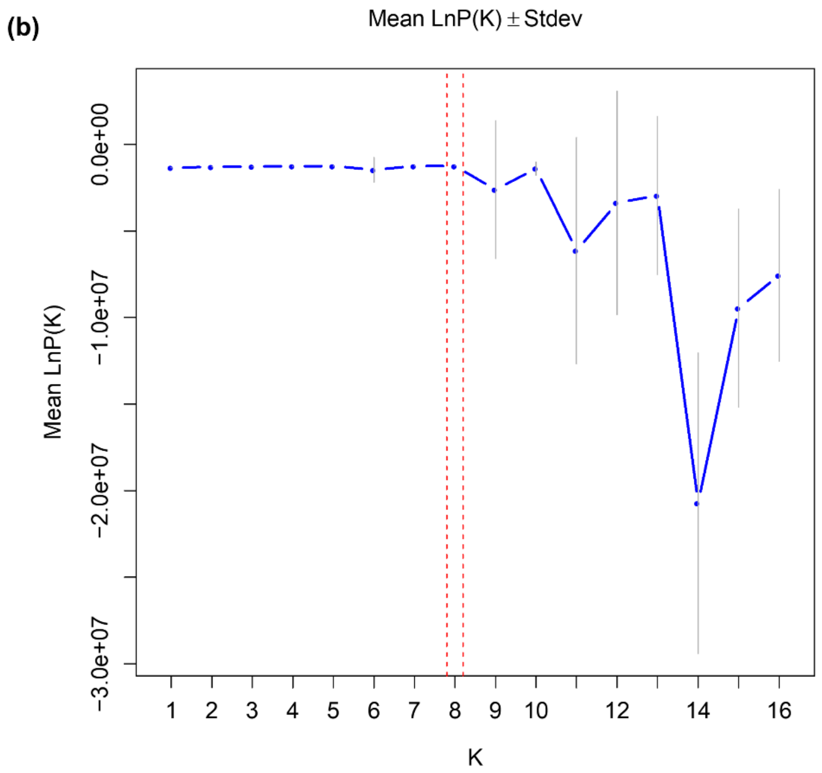

Figure S3. Structure analysis of *A. butcheri* from the southern regional cluster. Plots include (a) delta *k* (*Δk*) values for each predicted *k* and (b) the log likelihood of each predicted *k* (Mean LnP(*k*) ± Stdev).

K=1

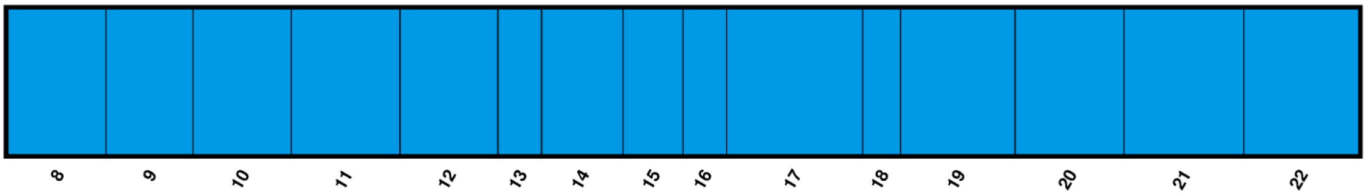
8
9
10
11
12
13
14
15
16
17
18
19
20
21
22

K=2

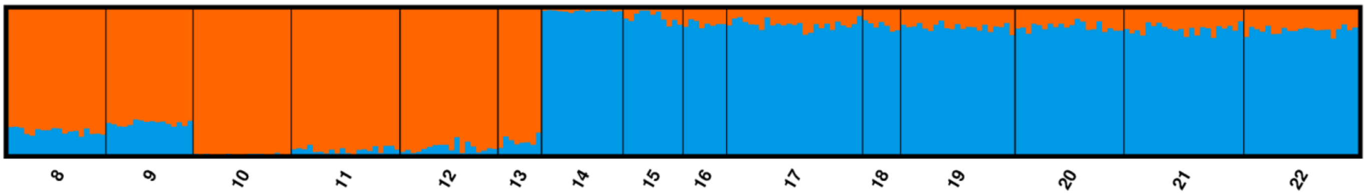
8
9
10
11
12
13
14
15
16
17
18
19
20
21
22

K=3

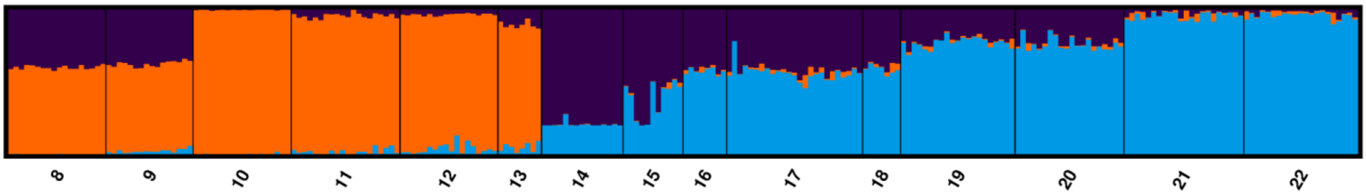
8
9
10
11
12
13
14
15
16
17
18
19
20
21
22

K=4

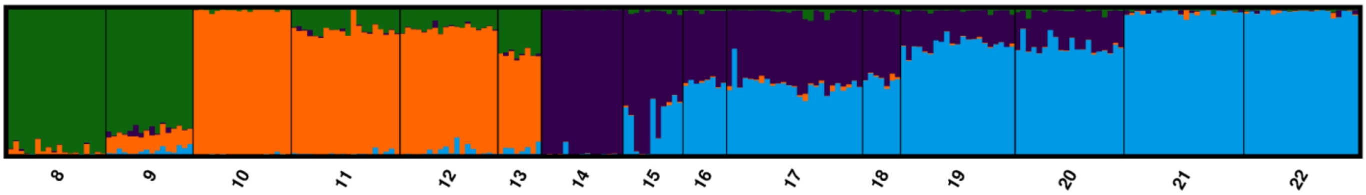
8
9
10
11
12
13
14
15
16
17
18
19
20
21
22

K=5

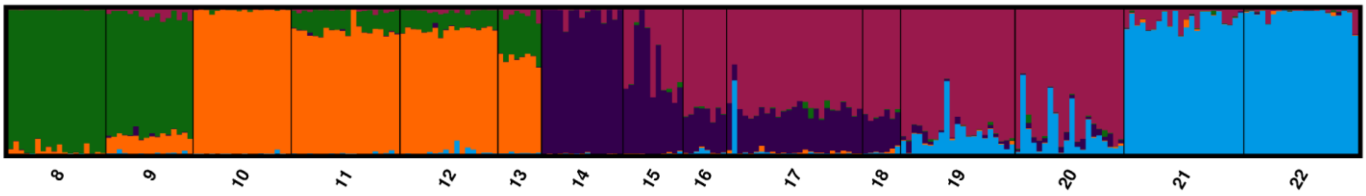
8
9
10
11
12
13
14
15
16
17
18
19
20
21
22

K=6

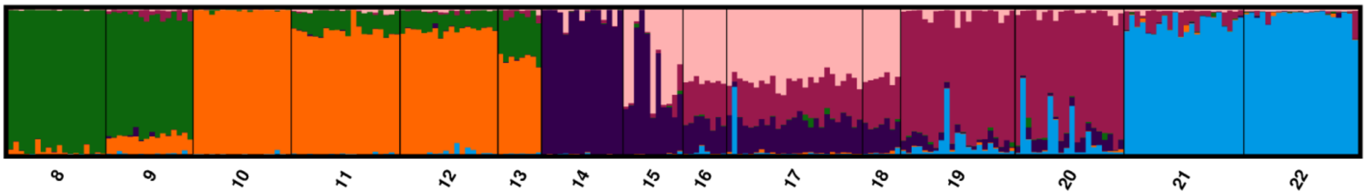

K=7

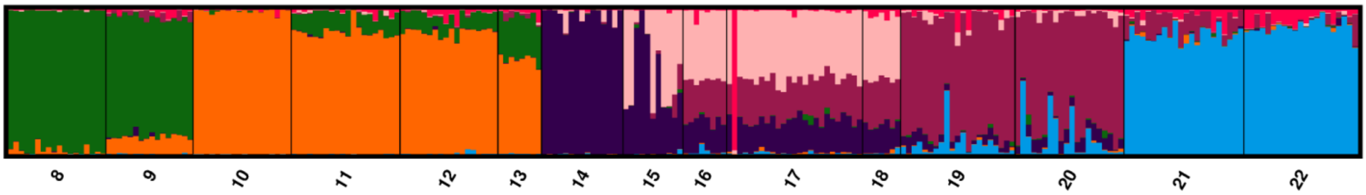

K=8

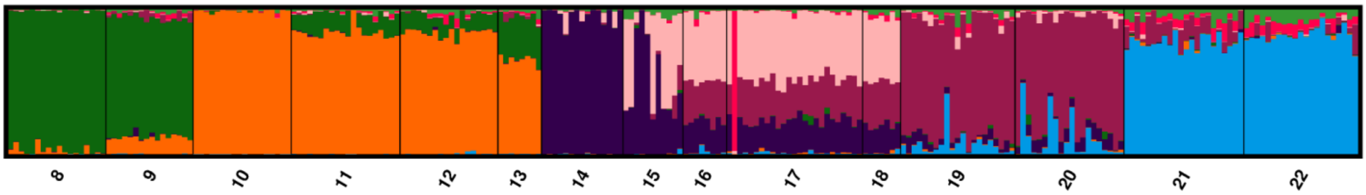

K=9

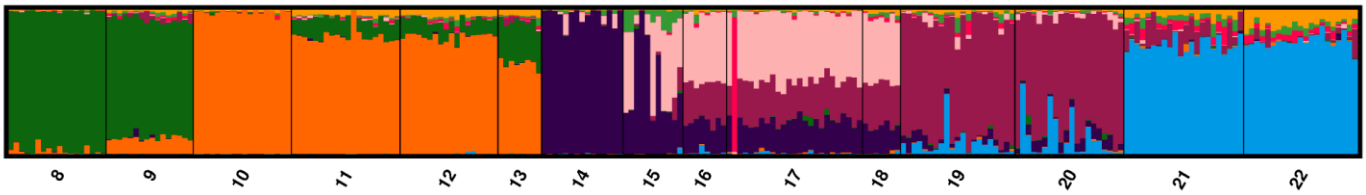

K=10

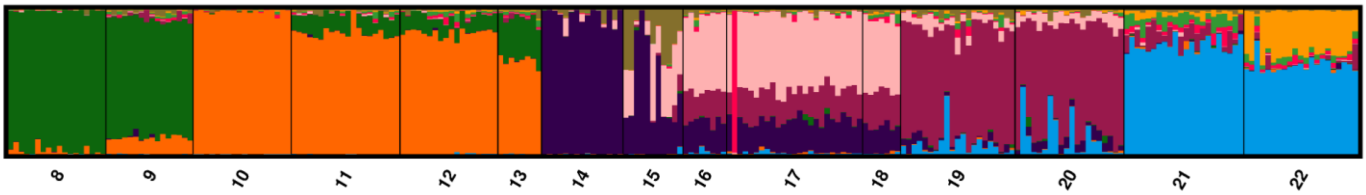

K=11

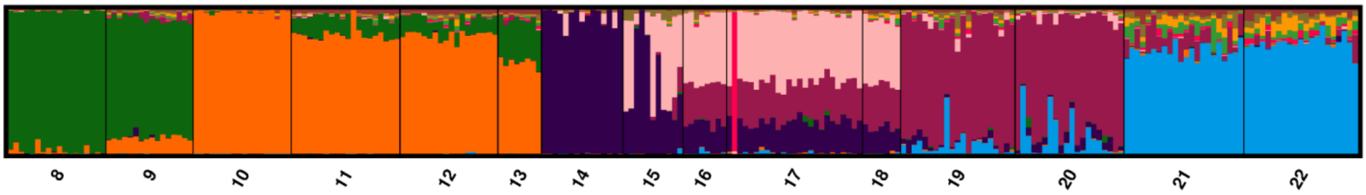

K=12

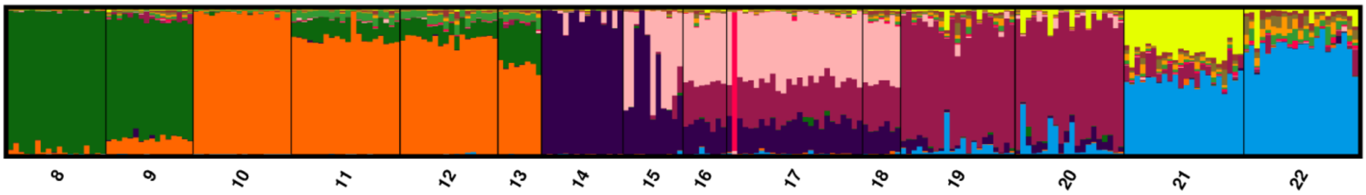

K=13

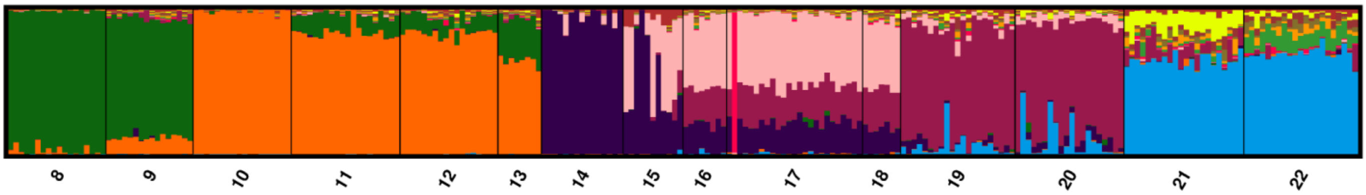

K=14

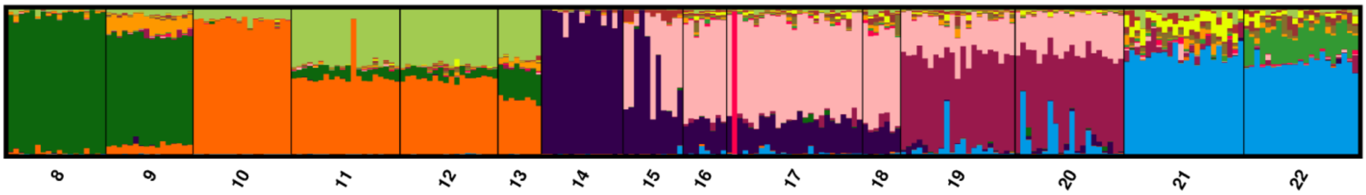

K=15

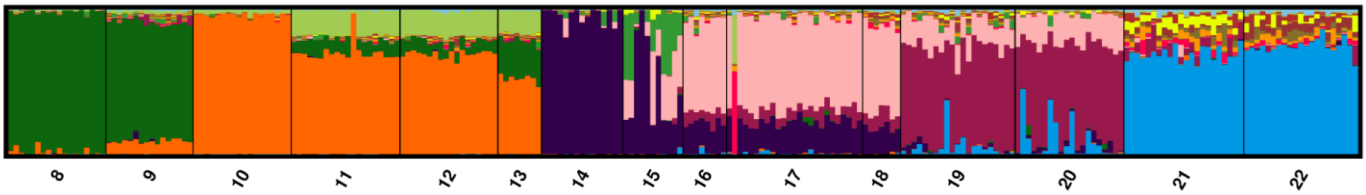

Figure S4. Structure plots for each predicted k (K=X) of *A. butcheri* from the southern regional cluster. Values below plots indicate capture location ID (refer to Table 1). Plot colours represent the predicted genetic cluster in each plot independenty.

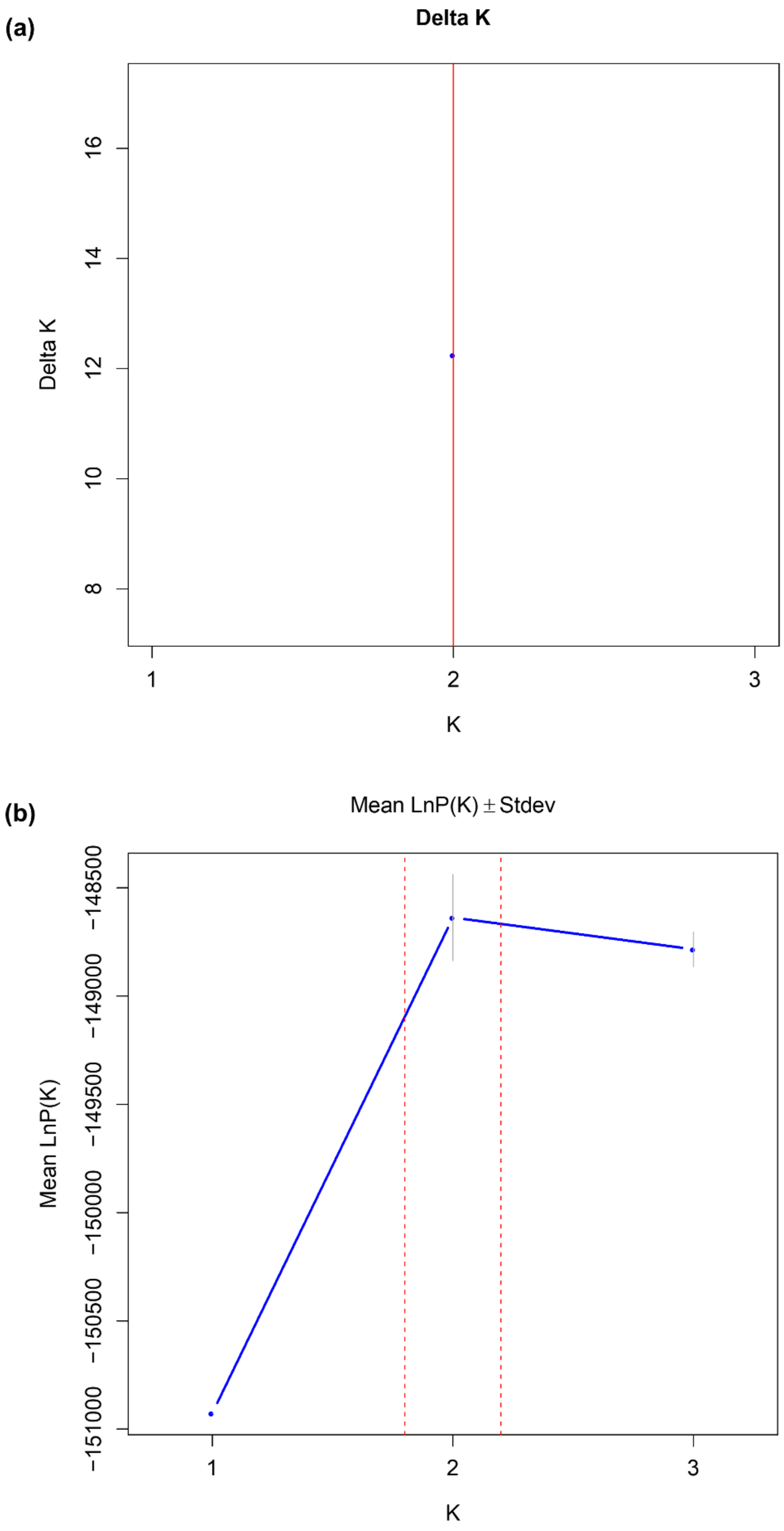

Figure S5. Structure analysis of *A. butcheri* from sub-cluster northKI. Plots include (a) delta *k* (*Δk*) values for each predicted *k* and (b) the log likelihood of each predicted *k* (Mean LnP(*k*) ± Stdev).

K=1

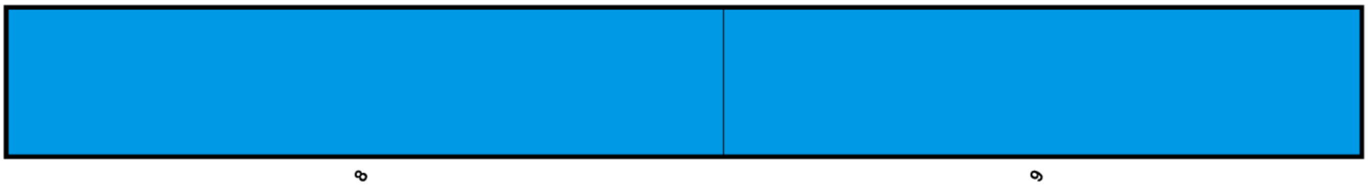

K=2

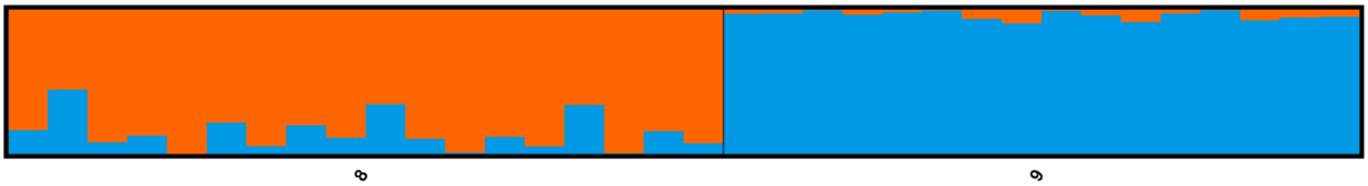

K=3

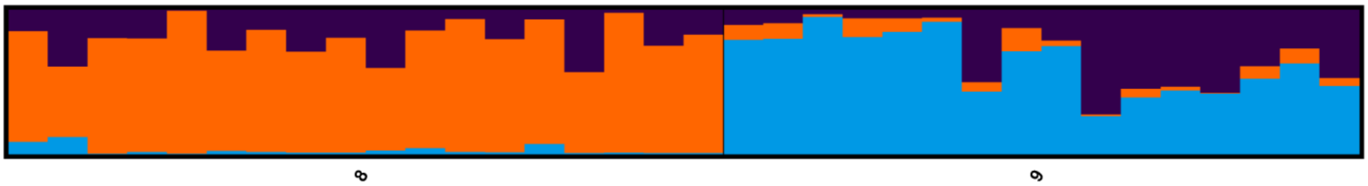

Figure S6. Structure plots for each predicted k (K=X) of *A. butcheri* from sub-cluster southKI. Values below plots indicate capture location ID (refer to Table 1). Plot colours represent the predicted genetic cluster in each plot independenty.

(a)

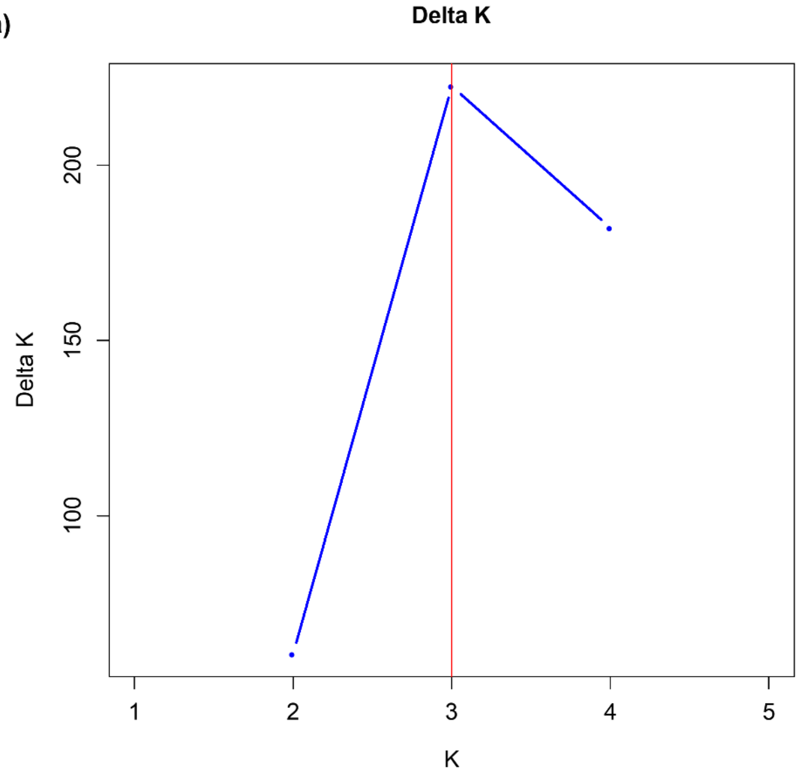

(b)

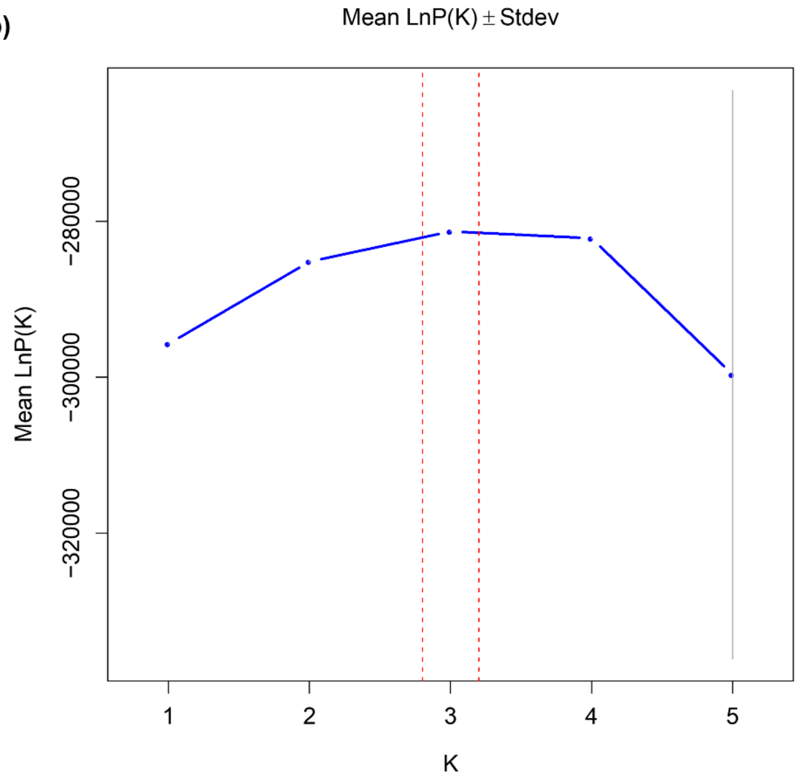

Figure S7. Structure analysis of *A. butcheri* from sub-cluster southKI. Plots include (a) delta *k* (*Δk*) values for each predicted *k* and (b) the log likelihood of each predicted *k* (Mean LnP(*k*) ± Stdev).

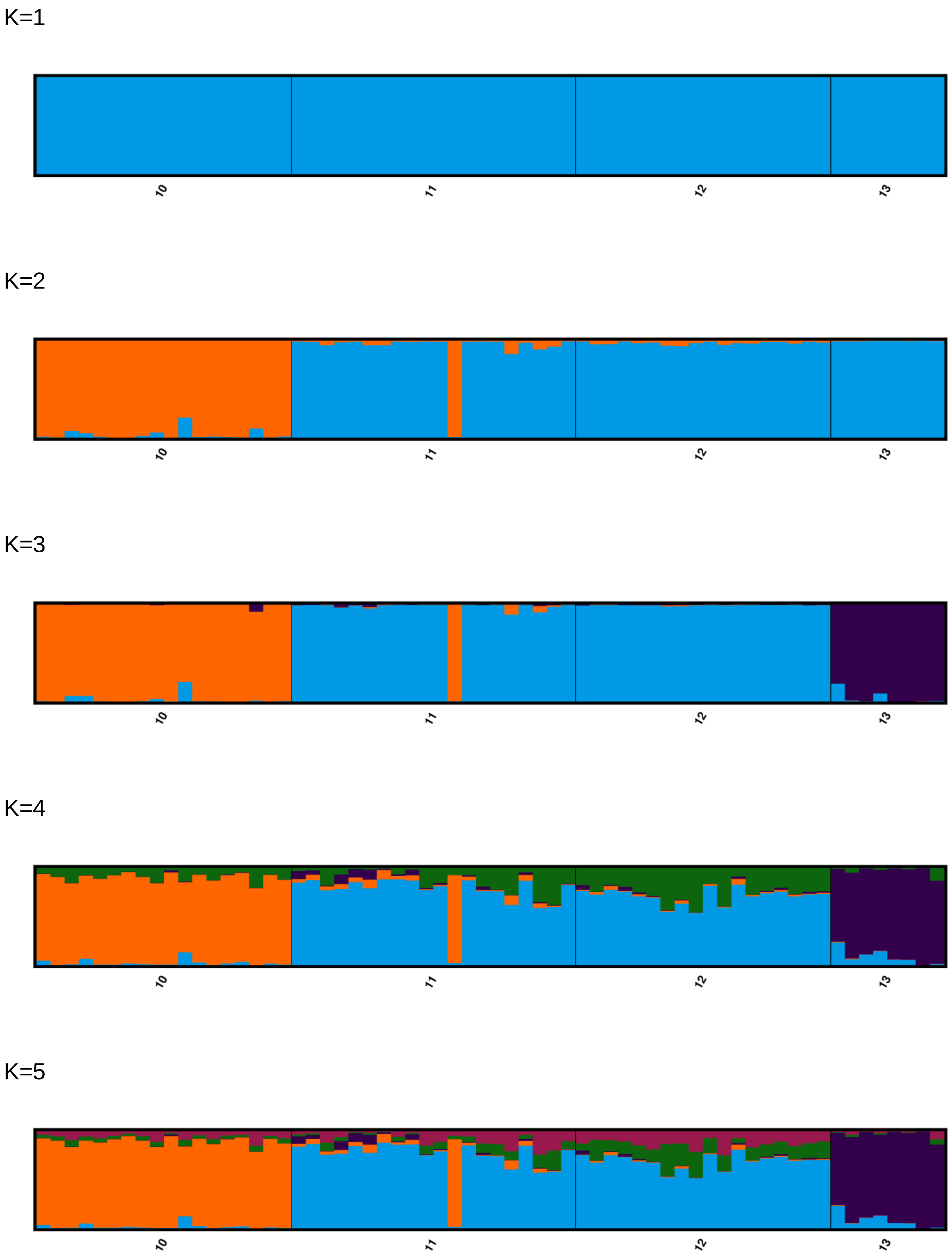

Figure S8. Structure plots for each predicted k (K=X) of *A. butcheri* from sub-cluster southKI. Values below plots indicate capture location ID (refer to Table 1). Plot colours represent the predicted genetic cluster in each plot independenty.

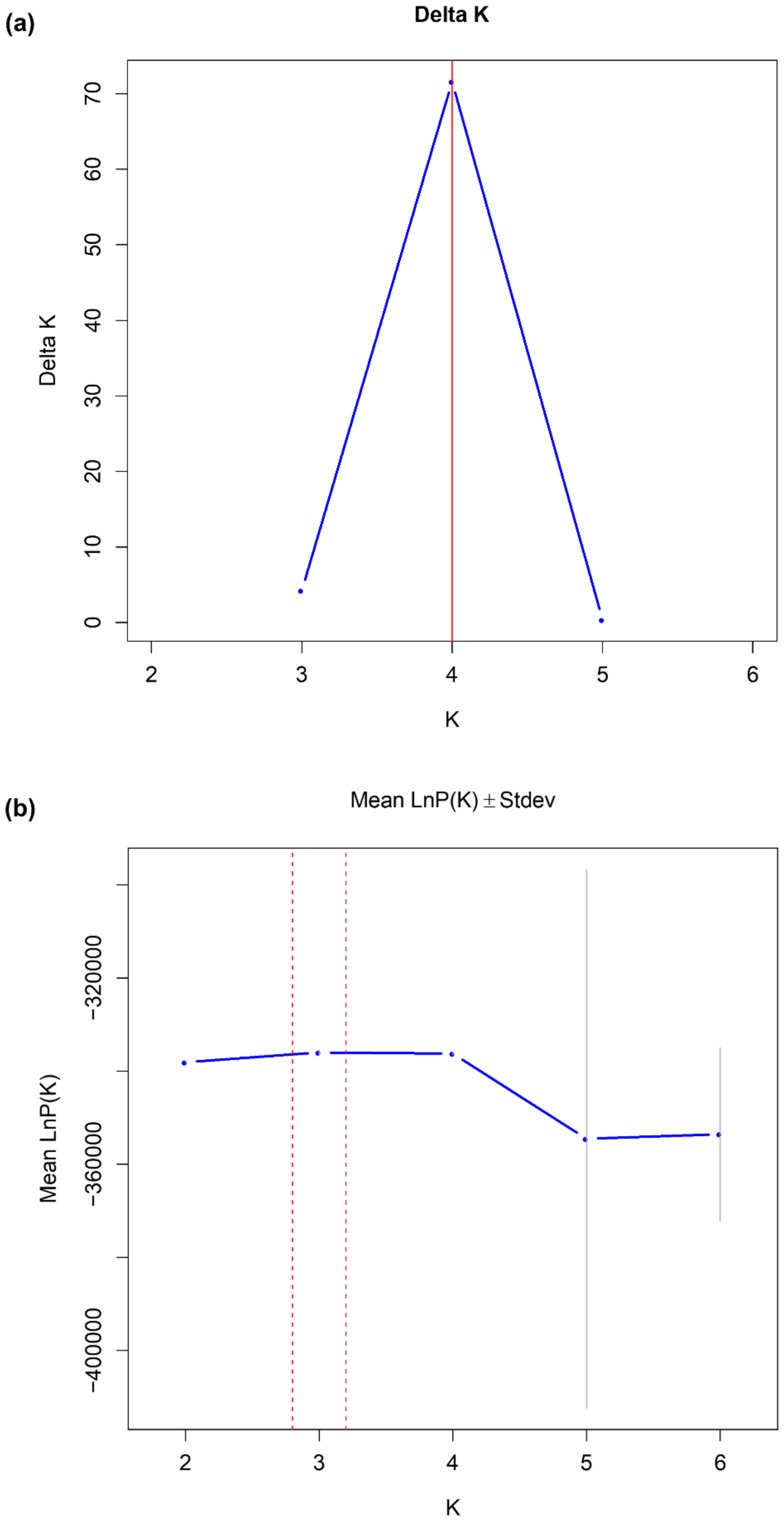

Figure S9. Structure analysis of *A. butcheri* from sub-cluster northSA. Plots include (a) delta *k* (*Δk*) values for each predicted *k* and (b) the log likelihood of each predicted *k* (Mean LnP(*k*) ± Stdev).

K=2

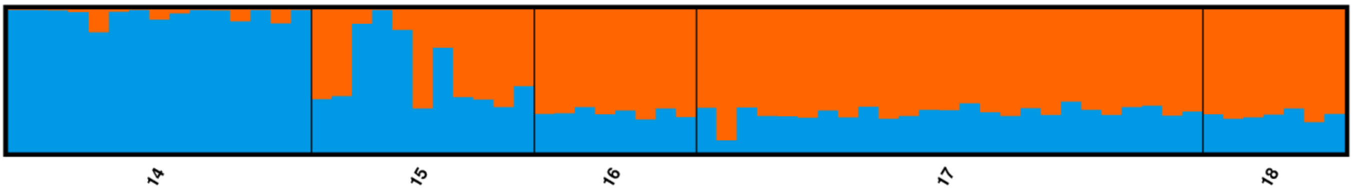

K=3

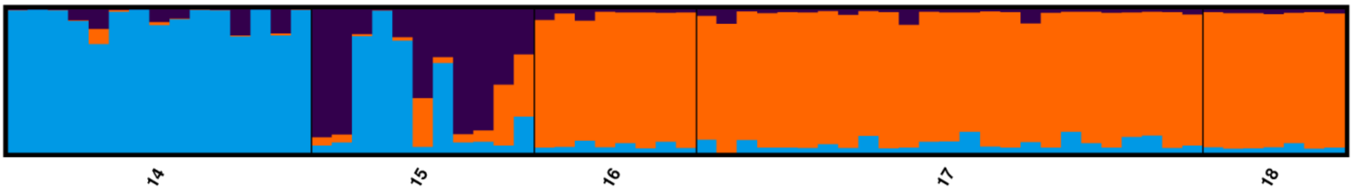

K=4

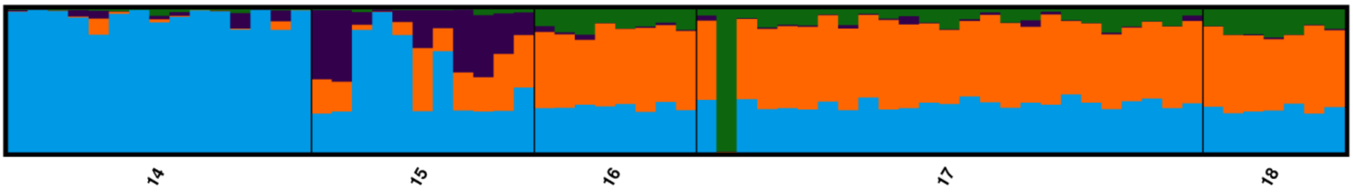

K=5

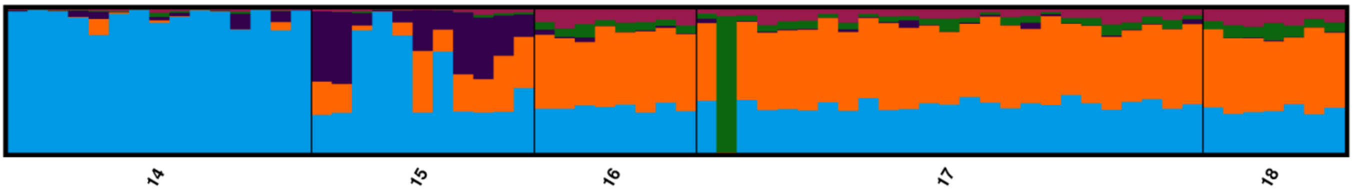

K=6

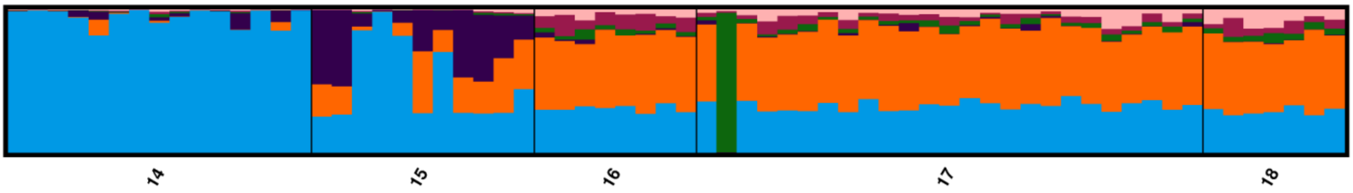

Figure S10. Structure plots for each predicted k (K=X) of *A. butcheri* from sub-cluster northSA. Values below plots indicate capture location ID (refer to Table 1). Plot colours represent the predicted genetic cluster in each plot independenty.

(a)

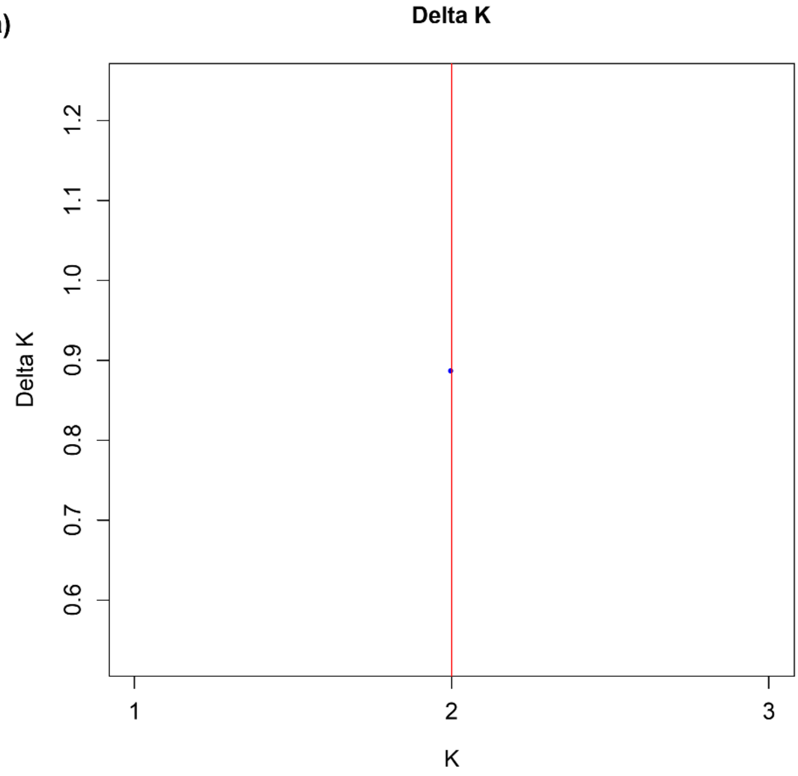

(b)

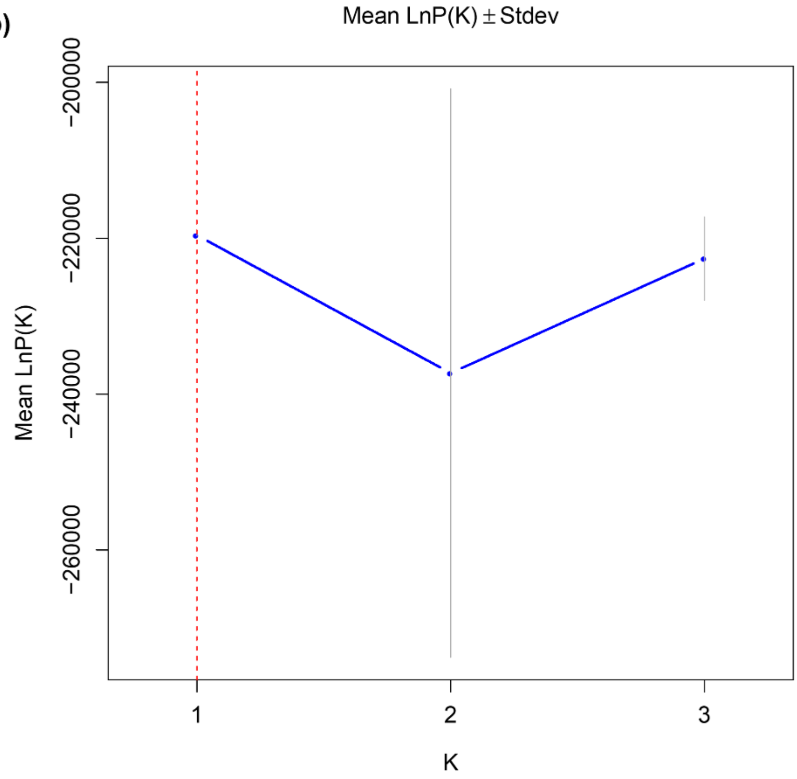

Figure S11. Structure analysis of *A. butcheri* from sub-cluster southSA. Plots include (a) delta *k* (*Δk*) values for each predicted *k* and (b) the log likelihood of each predicted *k* (Mean LnP(*k*) ± Stdev).

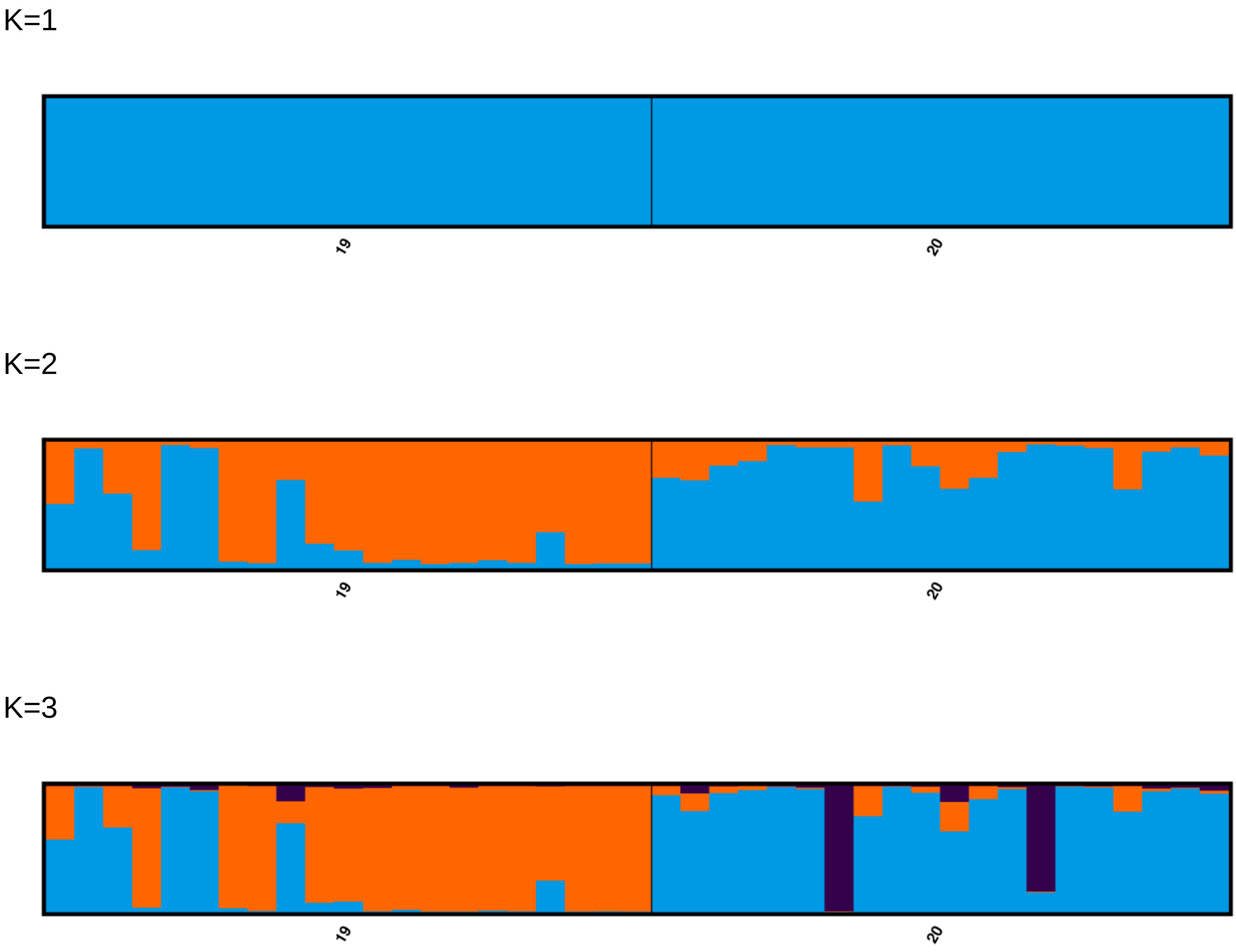

Figure S12. Structure plots for each predicted k (K=X) of *A. butcheri* from sub-cluster southSA. Values below plots indicate capture location ID (refer to Table 1). Plot colours represent the predicted genetic cluster in each plot independenty.

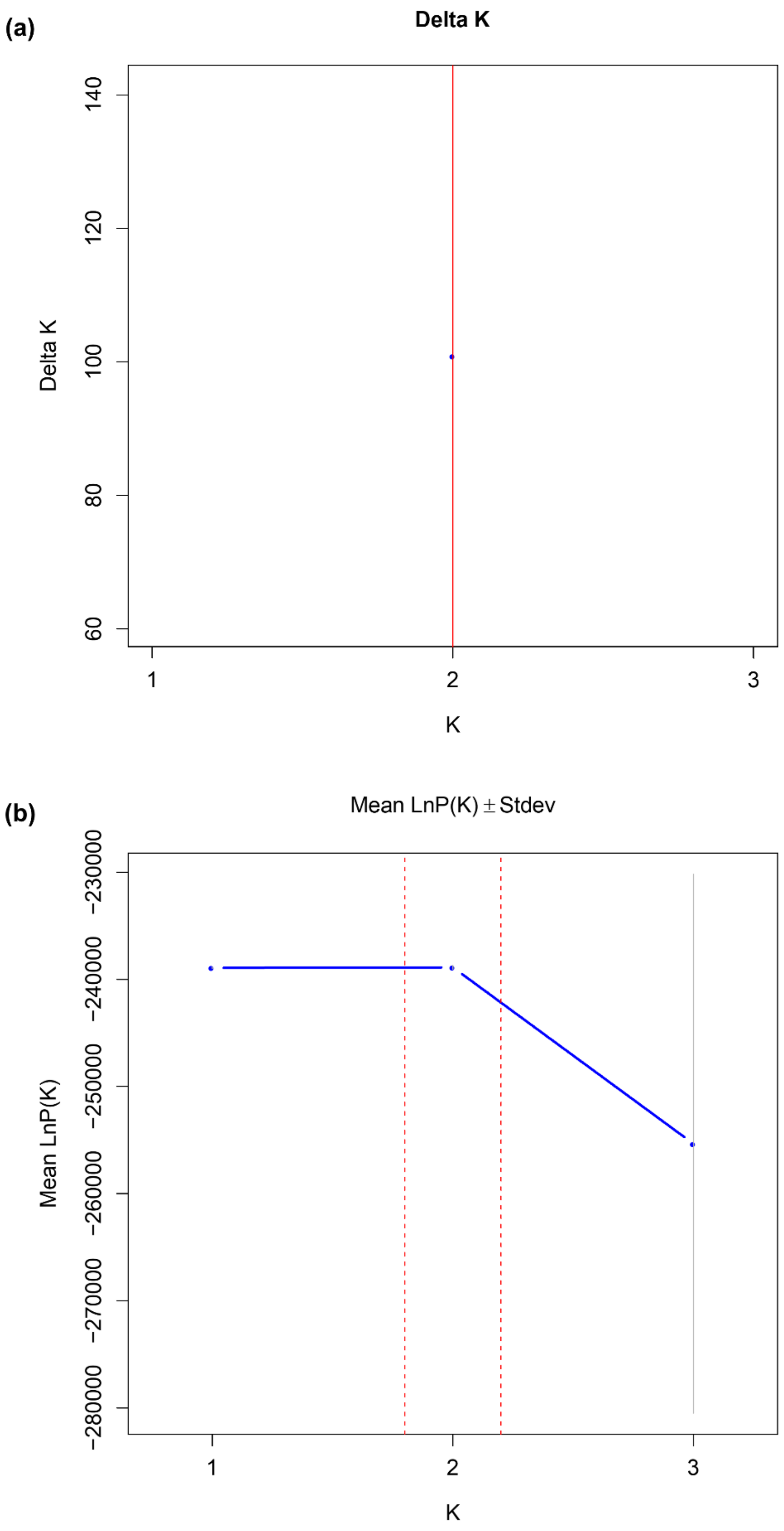

Figure S13. Structure analysis of *A. butcheri* from sub-cluster westVIC. Plots include (a) delta *k* (*Δk*) values for each predicted *k* and (b) the log likelihood of each predicted *k* (Mean LnP(*k*) ± Stdev).

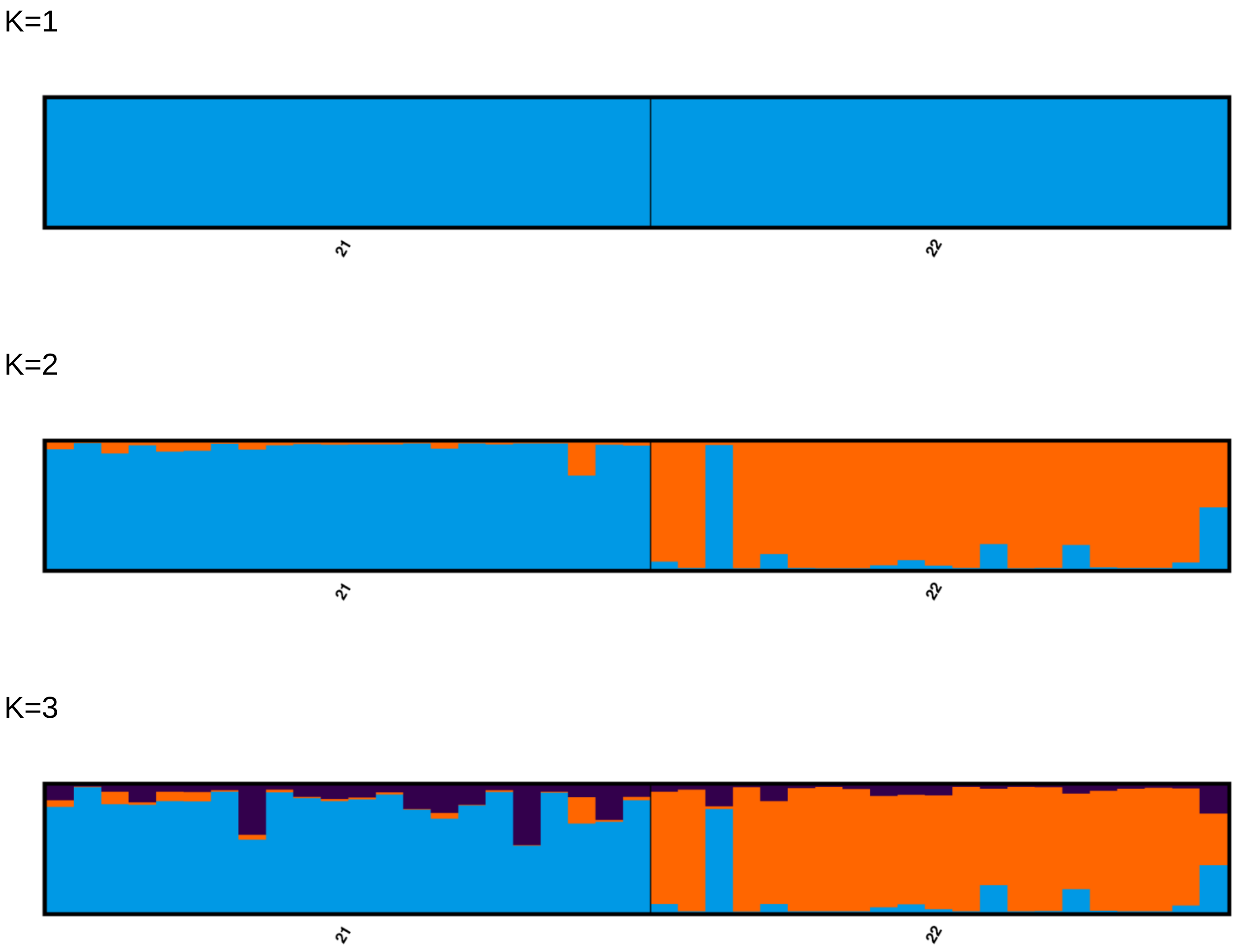

Figure S14. Structure plots for each predicted k (K=X) of *A. butcheri* from sub-cluster westVIC. Values below plots indicate capture location ID (refer to Table 1). Plot colours represent the predicted genetic cluster in each plot independenty.

Table S1. Summary of genotyped samples from each capture location for both *A. butcheri* and *A. australis* (*n* = 10). Table includes method of collection (collection type), coordinates, as well as fish length and weight averaged by capture location.

state	capture location		latitude	longitude	collection type	total length (mm)	body weight (g)
Western Australia (WA)	1	Swan River	-32.056440	115.729268	Net	99	17
	2	Collie River	-33.302336	115.667427	Mix	-	-
	3	Margaret River	-33.971249	114.985882	Mix	-	-
	4	Walpole-Nornalup	-35.005578	116.729803	Net	174	86
	5	Albany	-35.025274	117.937966	Rod	298	492
	6	Pallinup River	-34.469649	118.905147	Mix	-	-
	7	Bremer Bay	-34.391443	119.399468	Net	273	305
South Australia (SA)	8	Western River	-35.674364	136.971267	Net	124	35
	9	Middle River	-35.664786	137.073251	Net	150	66
	10	Stun Sail Boom	-36.020961	137.015023	Net	35	2
	11	Harriet River	-35.985951	137.181116	Mix	144	79
	12	Eleanor River	-35.975754	137.201555	Mix	118	59
	13	Chapman River	-35.787261	138.071934	Mix	150	55
	14	Port Lincoln	-34.740203	135.885176	Rod	279	355
	15	Tumby Bay	-34.376744	136.121845	Rod	323	611
	16	Port River	-34.786495	138.466801	Net	367	858
	17	West Lakes	-34.853334	138.498547	Net	228	195
	18	Onkaparinga River	-35.164248	138.466842	Rod	276	-
	19	Coorong	-35.564694	138.873332	Mix	362	697
	20	Robe	-37.159471	139.775441	Rod	323	512
Victoria (VIC)	21	Glenelg River	-38.063221	140.988287	Rod	312	-
	22	Hopkins River	-38.404822	142.509158	Rod	346	-
	23	Lake Tyers	-37.860443	148.091705	Rod	365	-
	24	Snowy River	-37.806754	148.558078	Rod	370	-
Tasmania (TAS)	25	Port Huon	-43.305684	147.120303	Rod	355	729
New South Wales (NSW)	26	Swan Lake	-35.201748	150.562453	Net	302	-
Queensland (QLD)	27	Nerang River	-27.933361	153.434958	Net	-	-

Table S2. Summary of number of loci retained after each quality control filter for the entire sampling distribution for both species, the *A. butcheri* distribution and the three regional clusters. Sample sizes included. Data for *A. butcheri* distribution and regional clusters excludes influential samples removed during data processing.

**step**	**action**	***A. butcheri* and *A. australis***	***A. butcheri distribution***	**western cluster**	**eastern *cluster***	**southern *cluster***
**0**	Raw data	33,493	33,493	33,493	33,493	33,493
**1**	Remove loci <95% reproducibility	19,991	20,613	18,677	21,116	21,800
**2**	Keep SNPs with data missing <20% overall individuals	19,991	20,613	18,677	21,116	21,800
**3**	Remove ambiguous SNPs - i.e., same position with more than two alleles	15,012	15,337	13,656	16,123	16,181
**4**	Remove monomorphic loci, including those with all NAs	15,012	12,096	6,796	10,458	8,893
sample size		449	435	110	77	248

Table S3. Pairwise comparison of *A. butcheri* samples with kinship coefficient values ≥ 0.20. Table includes both samples for each pairing, the proportion of SNPs with zero identity-by-state (IBS0) and kinship coefficient value (kinship). Samples removed indicated by '*'.

pair	sample ID 1	sample ID 2	IBS0	kinship
1	ABPhFT1-11*	ABPhFT1-13	0.0000	0.4692
2	ABHa3FT8-08*	ABHa3FT8-04	0.0001	0.4659
3	ABCh2FT8-01*	ABCh1FT8-01	0.0003	0.4593
4	ABSlFT1-19*	ABSlFT1-20	0.0009	0.4492
5	ABCoFT1-15	ABCoFT1-17	0.0087	0.2385
6	ABCoFT1-17	ABCoFT2-02	0.0063	0.2320
7	ABCoFT2-02	ABPaFT1-17	0.0100	0.2044

Table S4. A pairwise comparison of each capture location in the southern cluster based on genetic distance (FST). Locations are grouped by Australian state, including South Australia (blue) and Victoria (green), with further separation of South Australia into Kangaroo Island (light blue) and mainland Australia (dark blue). Colour key for FST range indicated below table.

Western River	Middle River	Stun'Sail Boom	Harriet River	Eleanor River	Chapman River	Port Lincoln	Tumby Bay	Port River	West Lakes	Onkaparinga River	Coorong	Robe	Glenelg River	Hopkins River	
0															Western River
0.0490	0														Middle River
0.1898	0.1630	0													Stun'Sail Boom
0.1273	0.1007	0.0854	0												Harriet River
0.1315	0.1039	0.0956	0.0049	0											Eleanor River
0.1440	0.1172	0.1524	0.0700	0.0709	0										Chapman River
0.1866	0.1593	0.2177	0.1555	0.1602	0.1836	0									Port Lincoln
0.1575	0.1287	0.1850	0.1253	0.1312	0.1505	0.0448	0								Tumby Bay
0.1399	0.1105	0.1703	0.1067	0.1123	0.1345	0.0672	0.0325	0							Port River
0.1262	0.1011	0.1512	0.0975	0.1015	0.1202	0.0604	0.0296	0.0023	0						West Lakes
0.1393	0.1095	0.1673	0.1045	0.1104	0.1321	0.0710	0.0353	0.0074	0.0054	0					Onkaparinga River
0.1295	0.1015	0.1522	0.0989	0.1017	0.1183	0.0847	0.0509	0.0267	0.0264	0.0261	0				Coorong
0.1281	0.0998	0.1525	0.0991	0.1026	0.1186	0.082	0.0496	0.0248	0.0242	0.0242	0.0065	0			Robe
0.1335	0.1074	0.1532	0.1032	0.1057	0.1219	0.0973	0.0657	0.0423	0.0419	0.042	0.0334	0.0311	0		Glenelg River
0.1328	0.1084	0.1541	0.1035	0.1068	0.1229	0.1006	0.0686	0.0473	0.0468	0.0469	0.0400	0.0367	0.0132	0	Hopkins River

FST

0 0.1 0.2
